# Glial receptor PLXNB2 regulates schizophrenia-related stress perception *via* the amygdala

**DOI:** 10.3389/fimmu.2022.1005067

**Published:** 2022-10-17

**Authors:** Fang-Ling Xuan, Ling Yan, Yanli Li, Fengmei Fan, Hu Deng, Mengzhuang Gou, Keerthana Chithanathan, Indrek Heinla, Liang Yuan, Kadri Seppa, Alexander Zharkovsky, Anti Kalda, L. Elliot Hong, Guo-Fu Hu, Yunlong Tan, Li Tian

**Affiliations:** ^1^ Institute of Biomedicine and Translational Medicine, Faculty of Medicine, University of Tartu, Tartu, Estonia; ^2^ Psychiatry Research Centre, Beijing Huilongguan Hospital, Peking University Health Science Center, Beijing, China; ^3^ Department of Medicine, Tufts Medical Center, Boston, MA, United States; ^4^ Maryland Psychiatric Research Center, Department of Psychiatry, University of Maryland, School of Medicine, Baltimore, MD, United States

**Keywords:** PLXNB2, microglia, astrocytes, amygdala, first episode schizophrenia, stress, anxiety

## Abstract

Stress is a trigger for the development of psychiatric disorders. However, how stress trait differs in schizophrenia patients is still unclear. Stress also induces and exacerbates immune activation in psychiatric disorders. Plexins (Plxn) and its ligands semaphorins (Sema) are important cellular receptors with plural functions in both the brain and the immune system. Recently, the role of Plxn/Sema in regulation of neuroinflammation was also noticed. Here, when investigating immune mechanisms underlying stress susceptibility in schizophrenia, we discovered the role of Plxnb2 in stress response. Patients of first-episode schizophrenia (FES) with high stress (FES-hs, *n*=51) and low stress (FES-ls, *n*=50) perception and healthy controls (HCs) (*n*=49) were first recruited for neuroimaging and blood bulk RNA sequencing (RNA-seq). A mouse model of chronic unpredictable stress (CUS) and intra-amygdaloid functional blocking of Plxnb2 were further explored to depict target gene functions. Compared to HCs, FES-hs patients had bigger caudate and thalamus (FDR=0.02&0.001, respectively) whereas FES-ls patients had smaller amygdala (FDR=0.002). Blood RNA-seq showed differentially expressed *PLXNB2* and its ligands among patient groups and HCs (FDR<0.05~0.01). Amygdaloid size and *PLXNB2* level were both negatively correlated with stress perception (*p*<0.01&0.05, respectively), which fully mediated the amygdaloid positive association with *PLXNB2* expression (β=0.9318, 95% CI: 0.058~1.886) in FES-hs patients. In mice, Plxnb2 was enriched in astrocytes and microglia and CUS reduced its expression in astrocytes (*p*<0.05). Inhibition of amygdaloid Plxnb2 by its functional blocking monoclonal antibody (mAb)-102 induced mice anxiety (*p*<0.05), amygdaloid enlargement (*p*<0.05), and microglial ramification (*p*<0.001) compared to saline. These data suggest that *PLXNB2* regulates amygdala-dependent stress responses.

## Introduction

Schizophrenia is synthesized by complex interactions of environmental factors with genetic predispositions, which burgeons in late adolescence-early adulthood (1, 2). Psychosocial stress disturbs brain homeostasis in susceptible subjects, altering neurotransmission and inducing inflammatory responses that exacerbates psychosis in schizophrenia (3, 4). Schizophrenia patients are variable in their sensitivity to psychosis-impacting stress, however, the underlying neurobiological mechanisms of which remain unelucidated but are proposed to be associated with genetic and epigenetic elements that encode brain cytoarchitectures consisting of both neuronal and non-neuronal cells in stress-regulating regions as well as their functional connectivity across the brain (3, 5–7).

Schizophrenia patients tend to show smaller brain volume in specific brain regions particularly involving the medial temporal limbic structures along with the white matter deficits, as demonstrated in magnetic resonance imaging (MRI) studies (8, 9). The hippocampus and amygdala are the most consistently reported regions that contribute to stress-induced psychosis and social withdrawal in both schizophrenia patients and animal models (7, 10). We recently reported that allostatic load, a composite physiological index of stress maladaptation including immune factors, negatively impacted cortical thickness and cognition (11) and was associated with enlargement of the choroid plexus – an important anatomical portal for brain-immune connection – in schizophrenia patients (12). This prompted us to further interrogate the immune-related molecular mechanisms of stress susceptibility in schizophrenia.

Plexins (PLXNs) are a family of cell surface receptors with key biological functions by binding to their ligands semaphorins (SEMAs) in multiple organs and are known to modulate the brain and behavior (13, 14). Of note, studies have shown their importance in the immune system as well (15). For instance, Plxnb2 has been shown to guide T cell migration to the germinal center to optimize antibody responses in the spleen (16). In the central nervous system, it regulates inflammatory pain (17) and microglia-mediated wound healing after spinal cord injury (18). These jointly imply the neuroimmune role of Plxn/Sema family in brain diseases.

Glial cells including astrocytes and microglia are the major inflammatory modulators in the brain, which regulate brain developmental processes and maintain brain structural and behavioral plasticity during adulthood (19–22). They are among the most sensitive biological targets of psychosocial stress and lever stress vulnerability-resilience *via* regulating neuroimmune crosstalk in neuropsychiatric disorders (7, 23). We for instance recently showed that microglia were important for stress adaptation in a mouse stress model (24).

Thus, we hypothesized that immune/glial receptors, particularly the Plxn/Sema family, may contribute to regulation of stress-induced neuroinflammation in schizophrenia. We hence first recruited a cohort of first-episode schizophrenia (FES) patients and healthy controls (HCs) to compare gene expressions in their peripheral blood cells, and we next examined Plxn/Sema expressions in mice undergoing chronic unpredictable stress (CUS) and further studied alterations of anxiety behaviors along with glial activation of mice after intra-amygdaloid functional blocking of Plxnb2 using a monoclonal antibody (mAb).

## Materials and methods

### Participants’ demographic and clinical measures

FES patients (*n*=101) were from the Beijing HuiLongGuan Hospital. Patients were diagnosed schizophrenia according to the Structured Clinical Interview for DSM-IV (SCID) independently by two psychiatrists. HCs (n=49) matched for age and sex were recruited from local community (**Table 1**). Candidates who unmet recruitment criteria were excluded. All participants provided written informed consent. The study was approved by the Institutional Ethical Committee of Beijing Huilongguan Hospital (No.2017-49).

**Table 1:** Demographic characteristics of FES patients and HCs.

Demographics	Mean (SEM)			F/*x^2^*	*P* (FDR)
	FES-hs	FES-ls	HC		
	(1)	(2)	(3)
	(*n*=51)	(*n*=50)	(*n*=49)	
**Gender (M/F)**	17/34	24/26	25/24	3.66	0.16
**Age (years)**	30.1 (1.51)	28.8 (1.07)	33.7 (1.40)	5.81	0.06
**Education (years)**	12.7 (0.49)	12.7 (0.45)	13.2 (0.33)	1.72	0.42
**Age of illness onset (years)**	28.4 (1.46)	28.0 (1.08)	NA	0.001	0.97
**Illness duration (months)**	16.0 (2.92)	10.0 (1.72)	NA	3.05	0.08
**Antipsychotic duration (days)**	5.4 (0.78)	4.0 (0.44)	NA	3.17	0.14
**Antipsychotic dosage (CPZ equivalent mg/day)**	232.8 (27.44)	271.6 (24.08)	NA	1.33	0.29
**Suicidality**	1.2 (0.08)	1.3 (0.09)	NA	0.59	0.44

All data were reported as mean (SEM); CPZ, Chlorpromazine; FDR, false discovery rate; FES-hs, first episode schizophrenia-high stress; FES-ls, first episode schizophrenia-low stress; HC, healthy control; Bold text indicates those with significant p or FDR value.

Participants’ past traumatic experiences were evaluated by Childhood Trauma Questionnaire (CTQ) of a retrospective measure encompassing five factors: physical abuse, emotional abuse, sexual abuse, physical neglect, and emotional neglect (25, 26). Participants’ stress levels were evaluated based on PSS questionnaire measuring feelings and thoughts during the past month (27, 28). Based on mean value of this score, patients were divided into two groups: high stress (FES-hs, PSS≥24) and low stress (FES-ls, PSS<24). Positive and Negative Syndrome Scale scores (PANSS) were measured independently by two psychiatrists (For details see **Supplementary Materials**).

### Whole blood collection and RNA-seq

Patient’s whole blood was collected after overnight fasting using PAXgene™ blood RNA tubes (Applied Biosystems). Total RNAs were extracted using Mag-MAX™ for Stabilized Blood Tubes RNA Isolation Kit (Applied Biosystems). RNAs were quantified and immediately sent to the Beijing Genomics Institute (BGI) for mRNA sequencing by BGIseq-500. Clean data of at least 20M reads per sample were collected. Data quality control and gene expression analysis were done on the Galaxy and NetworkAnalyst platforms (29) using EdgeR. Log2 fold changes (Log2FC) with the false discovery rate (FDR) were calculated for differentially expressed genes (DEGs) (For details see **Supplementary Materials**).

### Human and animal MRI

Human brain structural MRI data were acquired using a Siemens Prisma 3.0T MRI scanner with a 64-channel head coil. Sagittal images were collected following the ENIGMA protocol with FreeSurfer software (30). Intracranial volume (ICV) and regional volumes of bi- hemispheric structures were measured. Anesthetized mice (*n*=4+5) were scanned with 94/20 Bruker BioSpec small animal MRI. Coronal slices were obtained using a T2-weighted high-resolution sequence. The amygdaloid width was measured on slices in coordination with the anterior commissure using ImageJ (For details see **Supplementary Materials**).

### Animals and CUS

Fourteen 8-week-old C57BL/6NTac male mice (Taconic) were randomly assigned into control and CUS groups (*n*=7+7) and group-housed with free access to food and water under a standard 12 hours (h) light-dark cycle. Mice were exposed to a variable sequence of 7 mild and unpredictable stressors once per day for 5 consecutive weeks (31). For details see **Supplementary Materials**. Animal experiments were done under licenses No. 171 and No. 227 issued from the Estonian National Board of Animal Experiments.

### Intra-amygdaloid stereotaxic mAb microinjection

Twenty 10-12-week-old C57BL/6NTac male mice were deeply anesthetized with ketamine/xylazine (10/1.6mg/ml, 0.1ml/10g bodyweight, i.p.), the skulls were measured for amygdaloid coordinates (from Bregma: AP=-1.5mm, L= ± 3.0mm) and drilled open. Then, 0.5μl saline (*n*=10) or 10ng mAb102 in 0.5μl saline (*n*=10) was injected at each amygdaloid site with microinjection syringes that penetrated the brain at -4.5mm in depth. After surgery, animals were kept on a heating pad until fully awakened. Two-to-three animals were group-housed per cage after the operation.

### Open Field Test (OFT)

Five days (d) after microinjection, mice were habituated to ~400lux room light for 1h. Each individual mouse was measured for distance and time traveled in different zones of a digital box (44.8×44.8×45cm) *via* a software (Technical&Scientific Equipment GmbH) for 5min. The floor of the box was cleaned with 70% ethanol and dried thoroughly after each mouse.

### Elevated Plus Maze (EPM)

EPM consisted of open and closed arms (30×5cm each) intersected at a central 5×5cm square platform elevated to a height of 80cm. Mice were habituated to ~40lux room light for 1h. Individual mouse was placed on the central platform facing the open arm, and time spent on open or closed arms was recorded by a software (EthoVision XT, Noduls) for 5min. The arms were cleaned with 70% ethanol and dried thoroughly after each mouse.

### Flow cytometric analysis of brain cells

Mice (*n*=7+7 in both CUS and microinjection) were sacrificed with CO_2_. Tissues were gently homogenized through 70µm cell strainers (#352350, BD Biosciences) on ice as we described previously (24, 32). Homogenates were blocked in PBS+10% rat serum for 1h with gentle rotation at 4°C. Fluorescent antibody makers (0.5µl/marker, mostly from Biolegend) diluted in 200µl PBS+1%FBS were added and incubated for 1h at 4°C with light protection. For CUS experiment, Plxnb2-PE (#145903), CD11b-BV421 (#101251), CD45-BV650 (#103151), and Glast-APC (#130-123-555) were used. For microinjection experiment, CD11b-FITC (#101206), CD45-PE/Cy7 (#103114), Glast-PE (#130-118-344, Miltenyi), and MHCII-BV711 (#107643) were used. Washed samples were finally resuspended with FC buffer and filtered through 35μm cell strainers into flow tubes (#08-771-23, BD Biosciences). The acquisition was made with BD LSR Fortessa™ (BD Biosciences). Data were analyzed using Kaluza (Beckman Coulter). Percentages (%) of positively stained cells were calculated.

### Immunohistochemistry (IHC) and imaging

Anesthetized mice (*n*=3+3) were transcardially perfused with PBS+4% paraformaldehyde. Coronal sections of 40μm-thickness were washed in PBS and permeabilized in 0.5% triton X. Washed slices were incubated with primary antibodies including rabbit anti-IBA1 (#SKL6615, Wako, 1:500) and mouse anti-GFAP conjugated with AlexaFluor488 (#53-9892-82, eBioscience, 1:500) overnight at 4°C, followed by goat anti-rabbit IgG H&L-AlexaFluor568 antibody (#ab175471, Abcam, 1:500) and 0.1μg/ml DAPI (#ACRO202710100, VWR) and finally mounted to glass slides with Fluoromount™ aqueous mounting medium (#F4680, Sigma-Aldrich). Z-stack images were taken by a FV1200MPE laser scanning microscope at 60× resolution (Olympus). Cell morphometrics were quantified using ImageJ (For details see **Supplementary Materials**).

### Statistical analysis

Parametric methods (ANOVA and Student’s t-test) and nonparametric Mann-Whitney U test were used for continuous variables and chi-squared test for categorical variables in SPSS v27.0 (IBM). ANCOVA with age, gender, education, and ICV as covariates was conducted for human data. Mediation analysis was done using PROCESS v3.5 in SPSS. Figures were prepared in GraphPad Prism v8.0.1 and CorelDRAW Graphics Suite. Bonferroni method was used for *post hoc* pairwise comparison and multiple parameter comparisons were corrected by FDR. Data were presented as mean ± SEM, and *p* or FDR<0.05 was considered statistically significant.

## Results

### Perceived stress differed among FES patients and HCs

Since FES patients showed bimodal distribution in their perceived stress scale summation (PSSsum) scores, we divided them into two groups that had PSSsum scores above and below the average value of 24 derived from the whole patient cohort: e.g., FES patients both with high stress (FES-hs) and low stress (FES-ls). Specifically, FES-hs patients showed a higher PSSsum average score than both FES-ls patients and HCs (both FDR<0.001; **Table 2**), whereas FES-ls patients showed a lower PSSsum average score than HCs (FDR=0.03; **Table 2**). Gender distribution, age, and years of education did not significantly differ among patients and HCs (**Table 1**). Among patient groups, the age of illness onset, illness duration, and antipsychotic treatment did not differ (**Table 1**).

**Table 2:** Psychopathophysiological changes in FES patients.

Scores	Mean (SEM)			F/*x^2^*	*P* (FDR)	FDR	FDR	FDR
	FES-hs	FES-ls	HC			1 vs 2	1 vs 3	2 vs 3
	(1)	(2)	(3)
	(*n*=51)	(*n*=50)	(*n*=49)				
**CTQsum**	86.3 (5.36)	88.5 (5.11)	68.5 (4.54)	7.91	**0.02**	0.68	**0.03**	**0.04**
**PSSsum**	28.8 (0.31)	17.7 (0.91)	21.3 (0.64)	93.03	**< 0.001**	**< 0.001**	**< 0.001**	**0.03**
**PANSS-P**	21.8 (0.74)	22.2 (0.89)	NA	0.5	0.48			
**PANSS-N**	18.2 (0.90)	15.8 (0.79)	NA	2.74	0.1			
**PANSS-G**	39.9 (1.16)	35.1 (0.86)	NA	7.33	**0.01 (0.03)**			
**PANSS-T**	80.1 (1.94)	73.2 (1.66)	NA	4.13	0.05 (0.09)			

All data were reported as mean (SEM); CTQ, childhood trauma questionnaire; FDR, false discovery rate; FES-hs, first episode schizophrenia-high stress; FES-ls, first episode schizophrenia-low stress; HC, healthy control; PANSS, positive and negative symptom scale; P, positive symptom score; N, negative symptom score; G, general psychopathological symptom score; PANSS scores were controlled by age, sex, and education; PSS, perceived stress scale; Bold text indicates those with significant p or FDR value.

Notably, childhood trauma questionnaire summation (CTQsum) scores were higher in both patient groups compared to HCs (both FDR<0.05; **Table 2**), indicating more early life adverse experiences in patients. Furthermore, positive and negative symptom scale-general psychopathological symptom (PANSS-G) score was higher in FES-hs patients than in FES-ls patients (FDR=0.03; **Table 2**) and difference in PANSS-total (PANSS-T) was marginally significant (*p*=0.05; **Table 2**) while no differences were found for positive (PANSS-P) and negative (PANSS-N) symptoms. PSSsum scores were nominally correlated positively with PANSS-G scores in all FES patients (Spearman’s *r*=0.240, *p*=0.023, FDR>0.05) but not in separate patient groups. Suicidality records did not differ among patient groups, and no significant correlations between CTQsum and PSSsum or PANSS scores were found.

### Brain limbic structures differed in sizes among FES patients and HCs

**Figure 1:**
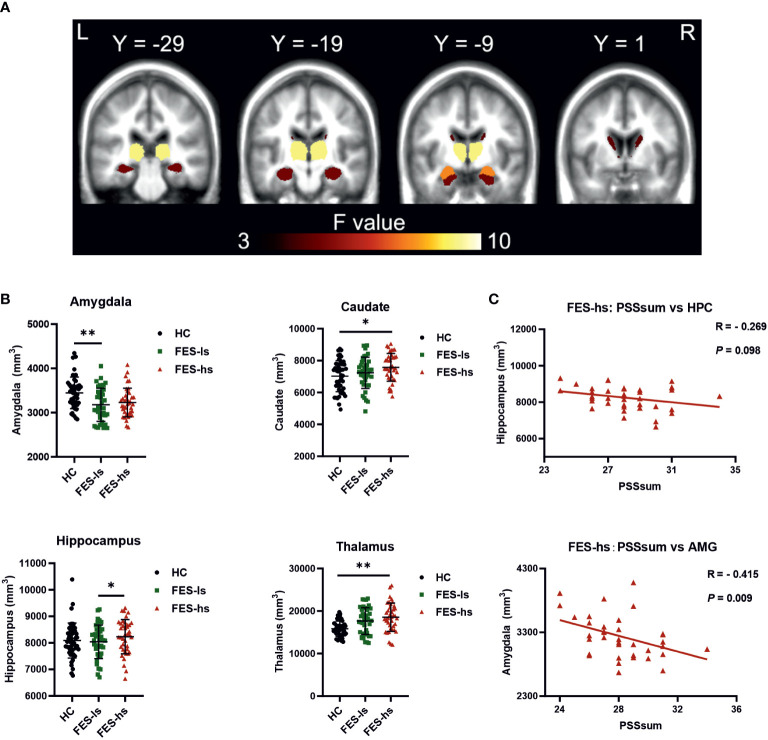
Brain subcortical limbic structures among FES patients and HCs. **(A)** Colors in MRI image represent ANCOVA *F* value of group differences. Yellow=thalamus, orange=hippocampus, red=amygdala, dark brown=caudate. **(B)** Volumes of the amygdala, caudate, thalamus and hippocampus were different among FES-hs (*n*=35) and FES-ls (*n*=36) patients and HCs (*n*=47), controlled by age, sex, education years, and ICV. *FDR<0.05, **FDR<0.01 (ANCOVA). **(C)** Correlations of sizes of the amygdala and the hippocampus with PSS scores in FES-hs patients (*n*=35, Spearman’s correlation). AMG: the amygdala; HPC: the hippocampus. See also **Table S1.1** and **Figure S1**.

Next, we measured the total brain intracranial volume (ICV) and volumes of subcortical limbic structures of FES patients and HCs by MRI (**Figure 1A** **)**. The ICV did not differ among patients and HCs after controlling age, gender, and education (**Table 3**). The amygdala, caudate, hippocampus, and thalamus exhibited significant group differences (**Figure 1B**; **Table 3**). Specifically, FES-hs patients had the largest volumes of caudate and thalamus, particularly compared to HCs (FDR=0.02&0.001, respectively; **Figure 1B**) and a larger hippocampal volume than FES-ls patients (FDR=0.01; **Figure 1B**). In contrast, smaller amygdala was found in FES patients, particularly in FES-ls patients (FDR=0.002; **Figure 1B**) and marginally so in FES-hs patients (*p*=0.07; **Table 3**) compared with HCs. No significant group differences were found in other subcortical limbic structures (**Table 3**). Antipsychotic dosages were not correlated with brain volumes in both patient groups.

**Table 3:** Volumes of brain limbic structures of FES patients and HCs.

Brain structure (mm^3^)	Mean (SEM)			*F*	*P* (FDR)	FDR	FDR	FDR
	FES-hs	FES-ls	HC			1 vs 2	1 vs 3	2 vs 3
	(1)	(2)	(3)
	(*n*=35)	(*n*=36)	(*n*=47)				
**Accumbens**	935.1 (18.86)	929.8 (28.79)	965.9 (25.73)	1.93	0.15			
**Amygdala**	3234.0 (56.22)	3180.6 (62.71)	3446.8 (51.98)	7.77	**0.001 (0.002)**	0.50	0.13	**0.002**
**Caudate**	7582.5 (150.95)	7023.4 (142.19)	7023.4 (974.84)	4.71	**0.01 (0.02)**	0.05	**0.02**	1.12
**Hippocampus**	8229.4 (102.55)	8014.5 (102.01)	8088.7 (96.45)	5.13	**0.007 (0.02)**	**0.01**	0.11	0.64
**Pallidum**	4194.5 (62.48)	4137.8 (98.36)	4150.2 (70.64)	1.13	0.33			
**Putamen**	10351.0 (158.35)	10494.4 (235.37)	10290.1 (200.17)	0.56	0.57			
**Thalamus**	18067.2 (502.66)	17449.9 (528.13)	15843.6 (259.93)	9.16	**< 0.001 (0.001)**	0.59	**0.001**	0.14
**ICV**	1534285.7 (23565.70)	1548888.9 (27581.85)	1549787.2 (24917.05)	0.26	0.77			

All data were reported as mean (SEM); FES-hs, first episode schizophrenia-high stress; FES-ls, first episode schizophrenia-low stress; HC, healthy control; FDR, false discovery rate; ICV, intracranial volume. ANCOVA was controlled by age, sex, education, and ICV and corrected for multiple comparisons among 7 regions; Bold text indicates those with significant FDR value.

### Negative correlations of amygdaloid size with perceived stress in high stress-FES patients

We further assessed correlations of the limbic structures with PSS scores and noted a negative relationship of amygdaloid size with PSSsum in FES-hs patients (*r*=-0.415, *p*=0.009, FDR=0.04; **Figure 1C**; **Table S1.1**) but not in other groups (**Figure S1B, S1C**). There was a trend of negative correlation between the hippocampal volume and PSSsum score in FES-hs group (*r*=-0.269, *p*=0.098). In contrast, both amygdaloid (*r*=0.31, *p*=0.03, FDR>0.05; **Figure S1C**) and hippocampal (*r*=0.419, *p*=0.003, FDR=0.01; **Figure S1C**) volumes were positively correlated with PSSsum in HCs.

Thalamic sizes were negatively correlated with PANSS-G scores in FES-hs patients while hippocampal sizes positively with PANSS-N scores in FES-ls patients (FDR=0.001&0.032, respectively; **Table S1.4**). No other significant correlations were found.

### *SEMA4*s and *PLXNB2* blood cell mRNA levels were higher in low stress-FES patients

To figure out what target immune genes may contribute to the limbic structural changes in FES-hs and FES-ls patients, we first explored peripheral blood cell RNA-seq data that we collected from a bigger cohort of FES patients and HCs earlier (33), which includes the subjects of our current study. In this cohort, out of the clustered DEGs using GO terms for molecular functions, PLXNs and SEMAs stood out as the top-ranking clustered protein domain with transmembrane signaling receptor activity (GO:0004888) (**Table S2.1, S2.2**). Notably, among the PLXN/SEMA family, *PLXNB2* and its ligands *SEMA4A/4B* were the most abundantly expressed members in the peripheral blood cells (**Table S2.3**).

**Figure 2:**
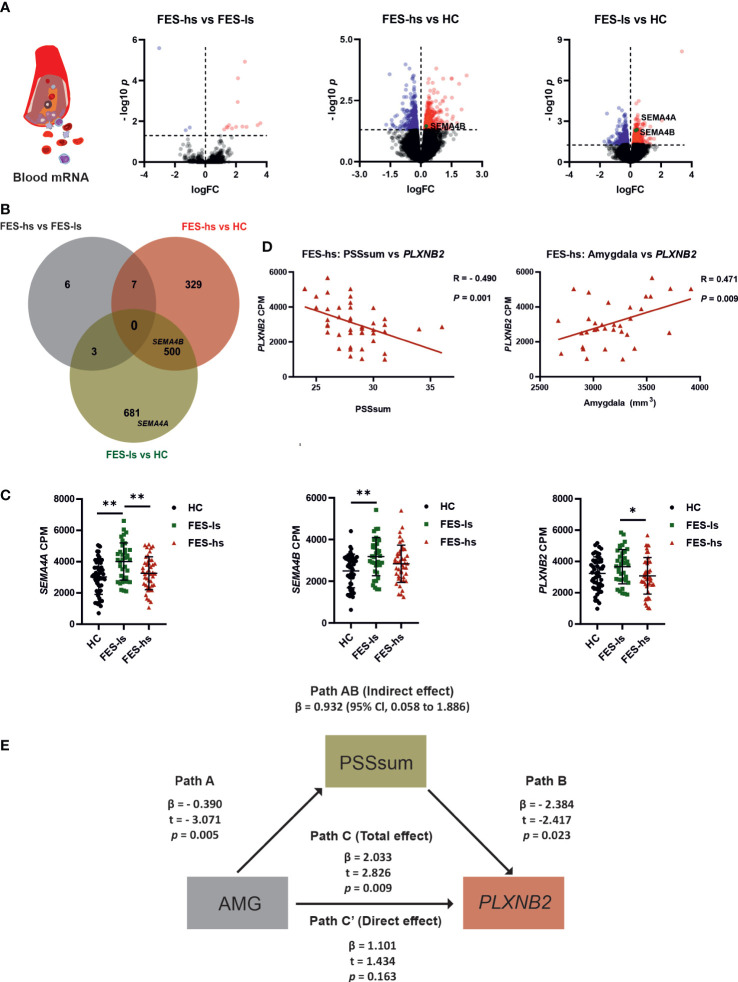
Increased SEMA4A, 4B and PLXNB2 mRNA levels and amygdaloid association with PLXNB2 expression mediated by stress perception in FES patients. **(A)** Volcano plots represent pairwise comparisons for RNA-seq DEGs in peripheral blood of FES-hs (*n*=44) and FES-ls (*n*=37) patients versus HC (*n*=48) individuals. Genes with FDR<0.05 are colored (red: up-regulation; blue dots: down-regulation). *SEMA4A* and *SEMA4B* are highlighted. **(B)** Venn diagram illustrates overlapping and group-specific DEGs. Circles denote genes, overlapping regions between circles denote respective shared genes among the groups. The upper histogram chart represents numbers of pairwise DEGs and the lower histogram chart represents numbers of overlapping and nonoverlapping DEGs. **(C)** Blood mRNA levels of *SEMA4A*, *SEMA4B* and *PLXNB2* in HCs, FES-ls and FES-hs patients, controlled by age, sex, and education. **(D)** Correlations of *PLXNB2* with PSSsum (Spearman’s, *n*=44) and with amygdaloid size (Pearson’s, *n*=28) in FES-hs patients, controlled by age, sex, and education. **(E)** PSS mediated positive amygdaloid association with *PLXNB2* in high-stress FES patients (Path AB), controlled by age, sex, and education. Path A represents a direct negative amygdaloid relationship with PSSsum. Path B represents a direct negative PSSsum with *PLXNB2*. Path C’ represents a direct amygdaloid relationship with *PLXNB2* while path C represents a positive amygdaloid with *PLXNB2* when PSSsum was considered, indicating a full mediation effect of PSSsum. *FDR<0.05, **FDR<0.01 (ANCOVA). See also **Tables S1.2, S1.3** and **Figure S1**.

We further repeated DEG analysis on the available RNA-seq counts of from subjects of our current cohort (FES-hs=44, FES-ls=37, HC=48), which showed in total 1247 DEGs with upregulated *SEMA4A* and *SEMA4B* among them in FES-ls and FES-hs groups (**Figure 2A**; **Table S3.1**). Compared to HCs, 1184 (472 up-regulated and 712 down-regulated) and 836 (405 up-regulated and 431 down-regulated) DEGs were obtained in FES-ls and FES-hs patients, respectively, while only 16 DEGs were differentiated between the two patient groups (**Figure 2B**). Group-wise comparisons also noted 500 overlapping DEGs including *SEMA4B* shared by both FES-hs and FES-ls patients as compared to HCs, whereas *SEMA4A* was upregulated only in FES-ls patients (**Figure 2B**). Pathway analysis showed that the 500 overlapping DEGs were mostly related to cancer biology and cell proliferation, while the FES-hs-specific 329 DEGs involved a small group of cytokine signaling genes and the FES-ls-specific 681 DEGs were engaged with leukocyte activation and innate immune response (**Table S3.2**). Between the two patient groups, the 6 nonoverlapping DEGs were related to hemoglobin and glycophorin a (**Table S3.2**). Although *PLXNB2* didn’t show up in the 3 clusters of DEGs, it was differentially expressed between FES and HC groups in the bigger cohort (FDR=0.007, **Table S2.3**).

To exclude the confounding effects of age, gender and education on gene expression, we further controlled these factors in group-wise comparisons and observed consistent upregulations of *SEMA4A* and *SEMA4B* (**Figure 2C**). It is noteworthy that their receptor *PLXNB2* was also upregulated in FES-ls patients compared with FES-hs patients (FDR=0.046; **Figure 2C**). No significant changes in other *SEMA* and *PLXN* receptors were observed (**Table S3.1**). We next pursued the associations of PLXN/SEMA expressions with PSS scores and brain volumes.

### PSS mediated positive amygdaloid association with blood PLXNB2 expression in high stress-FES patients

By incorporating the RNAseq, MRI, and symptom data, we found that blood mRNA level of *PLXNB2* was negatively correlated to PSSsum in all FES patients (*r*=-0.337, *p*=0.002; **Figure S2A**), especially in FES-hs patients (*r*=-0.490, *p*=0.001, FDR=0.003; **Figure 2D**; **Table S1.2**), but not in FES-ls and HCs (**Figure S2B & S2C**). Conversely, there was a significant positive correlation between *PLXNB2* and amygdaloid volume in combined FES patients (*r*=0.228, *p*=0.035; **Figure S2E**) and in FES-hs patients (partial *r*=0.471, *p*=0.009, FDR=0.04; **Figure 2D**, **Table S1.3**) after controlling age, gender, and education, but not in FES-ls and HCs (**Figures S2F, S2G**; **Table S1.3**). Antipsychotic dosages were not correlated with *PLXNB2* levels in both patient groups and no significant correlations with PSS and PANSS scores were found on *SEMA4A* and *SEMA4B* (**Table S1.2, S1.5**).

As blood *PLXNB2* expression could be a sensitive molecular substrate of stress, which is regulated by the amygdala, we performed a mediation analysis predicting that brain structural deficit underlies the stress-induced *PLXNB2* expressional changes in FES patients, e.g., amygdaloid volume as the independent variable, *PLXNB2* mRNA level as the dependent variable, and PSSsum as the mediator, controlled by age, gender, and education (**Figure 2E**). The direct effect on the association of amygdaloid volume with *PLXNB2* as denoted by Path C’ was insignificant, whereas the total effect on the association as denoted by Path C, when PSS was introduced as a mediator (path AB: β=0.9318, 95% CI: 0.058~1.886), was significant (*p*=0.009, **Figure 2E**). As a result, the model suggested a positive effect of the amygdala on *PLXNB2*, which was fully mediated by PSSsum in FES-hs patients. However, no such effects existed in FES-ls or HCs and neither for *SEMA4A* and *SEMA4B*. To further understand the role of Plxnb2 in stress response and the underlying brain mechanism, we next investigated with animal models.

### Plxnb2 was enriched in glial cells and decreased by CUS in mice

**Figure 3:**
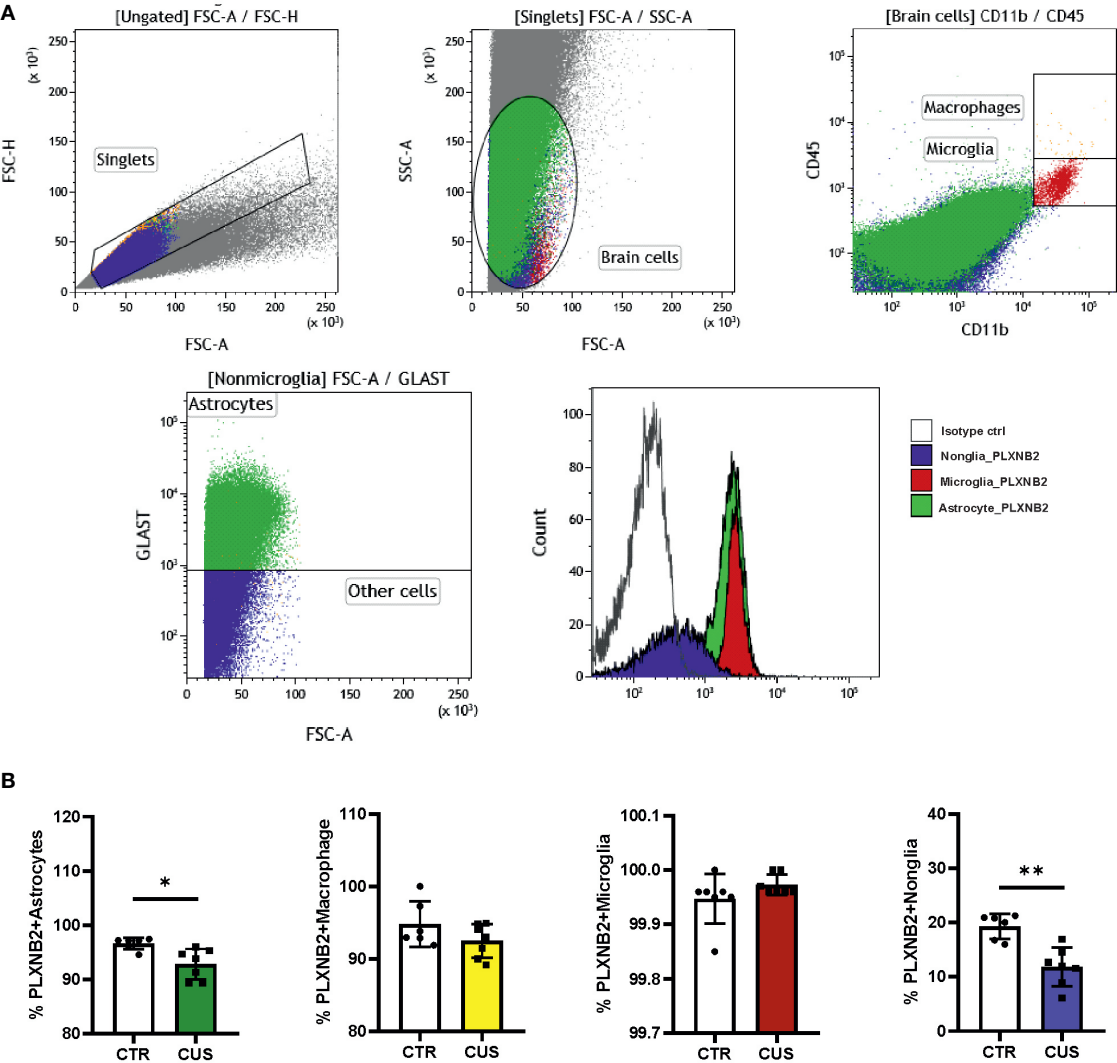
Mouse Plxnb2 expression in glial and nonglial cells after chronic unpredictable stress (CUS). **(A)** Flow cytometric staining of brain cells in the hippocampus is illustrated in representative gating dot plots. Cell populations stained by Plxnb2-specific antibody and isotype antibody are shown in overlaid histograms. **(B)** Populations of Plxnb2-expressing astrocytes, macrophage, microglia and nonglial cells were compared. Plxnb2 was enriched in glia and showed decrease in CUS group (*n*=7 mice) compared to the control (CTR) group. *p<0.05; **p<0.01 (Student’s t-test). See also **Figure S3**.

We first explored available brain transcriptomic data. Online brain RNA-seq databases show the enrichment of *PLXNB2* in human/murine microglia as compared to other brain cell types (**Figure S3A**). We also explored a GEO dataset on isolated murine microglia from different developmental stages (34), showing that *Plxnb2* was stably expressed in microglia from E16.5 onward (**Figure S3B**). Besides, murine astrocytes also highly express *Plxnb2* (**Figure S3C**). To confirm this and to understand the stress effect on *PLXNB2* in the brain, we next studied Plxnb2 expression in a CUS mouse model using specific markers for astrocytes, microglia, and macrophages in flow cytometry (**Figure 3A**). Compared to immunohistochemistry, flow cytometry allowed us to quantitatively measure different brain cell types and Plxnb2 expression more efficiently. Plxnb2 was enriched in hippocampal glial cells, especially in astrocytes and microglia (**Figures 3A, B**), but much less so in nonglial cells (e.g., neurons).

In CUS, no significant interaction between CUS and cell type was observed (data not shown) but there was a main effect of CUS (*p*=0.004) (*n*=7+7). Plxnb2 expression in astrocytes (*p*=0.020; **Figure 3B**) and nonglial (*p*=0.004; **Figure 3B**) were decreased after CUS compared to control, whereas no changes were observed in microglia and macrophages (**Figure 3B**). Considering that astrocytes and nonglia represent the great majority of adult brain cells, this result indicates that Plxnb2 was overall downregulated in the brain by CUS. We also measured *Sema4a* and *Sema4b* mRNA levels in the CUS model but did not find significant changes in them (data not shown).

### Plxnb2 blocking in the amygdala induced anxiety-like behavior in mice

Based on the above data, we postulated that Plxnb2 in the amygdala may regulate stress response or anxiety. To test this, we selected a blocking antibody (mAb102) that targets extracellular epitope of both human and mouse PLXNB2 with high affinity. The EC50s for mAb102’s binding to CHOK1 cells expressing human and mouse *PLXNB2* were 1.0nM and 2.2nM, respectively (**Figure S4A**). Functionally, mAb102 was able to inhibit the binding of angiogenin (ANG) and SEMA4C, the two ligands of PLXNB2, to CHOK1-*PLXNB2*, with an IC50s of 10.6 and 13.2nM, respectively (**Figure S4B**).

**Figure 4:**
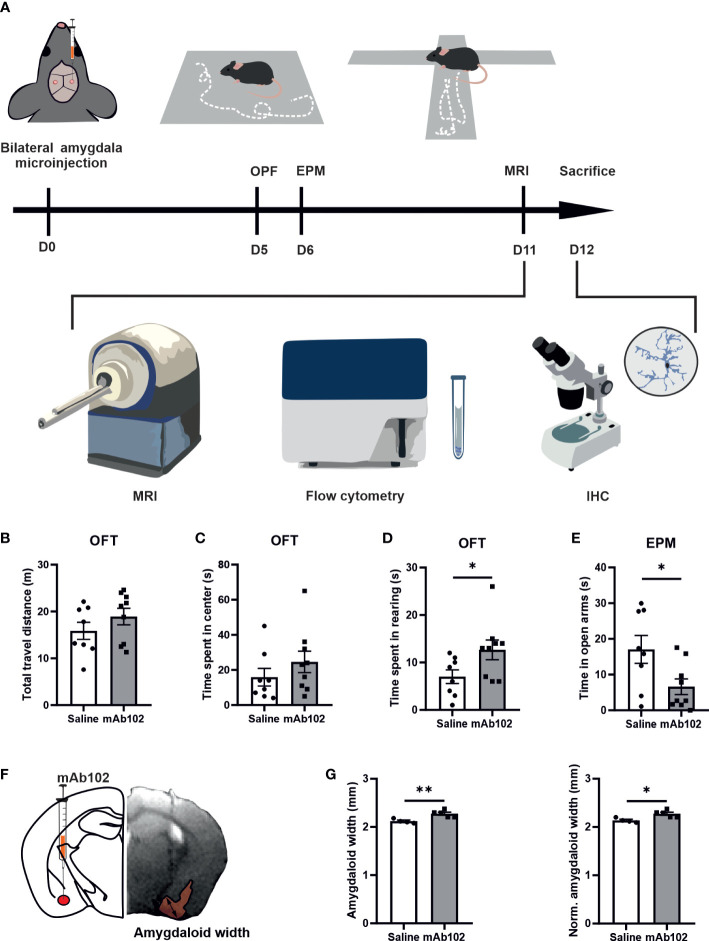
Anxiety-like behaviors in mice and quantification of amygdaloid width. **(A)** Schematic diagram of experimental design. 0.5 μl mAb102 (10 ng, *n*=9 mice) or saline (*n*=8 mice) was injected in each amygdala of mice bilaterally and open field test (OFT) and elevated plus maze (EPM) test were conducted on the 5th and 6th day after the operation, respectively. Mice were later sent for magnetic resonance imaging (MRI) scan. The brains were collected for flow cytometry and immunohistochemistry (IHC). **(B–D)** In OFT, total travel distance, time spent in the central zone and in rearing by mice highlighted anxiety-related exploration of mAb102-treated mice. **(E)** Time spent in EPM open arms indicated enhanced anxiety in mAb102-treated mice. See also **Figure S4**. **(F)** Intra-amygdala injection site is illustrated in representative brain coronal diagram and the corresponding MRI image of injection is shown, with the measured amygdaloid width indicated. **(G)** Amygdaloid width before and after normalization with the maximal brain width showed increase in mAb102 group (*n*=5 mice) compared to saline group (*n*=4 mice). *p<0.05; **p<0.01 (Student’s t-test).

We injected 10ng of mAb102 or saline into each amygdala per mouse (*n*=10+10). Five days later, we studied anxiety-like behaviors in OFT and EPM, followed by alive animal brain MRI scan, and by flow cytometry and IHC upon animal sacrifice (**Figure 4A**).

In OFT, total travel distance showed no group difference, indicating equivalent locomotor activity among treated animals (**Figure 4B**). While time spent in the central zone had no group difference (**Figure 4C**), time spent in rearing was longer in mAb102-treated mice than in saline-treated mice (*p*=0.04; **Figure 4D**). Moreover, mAb102 group showed shorter time spent on open arms in EPM test (*p*=0.039; **Figure 4E**).

### Plxnb2 blocking in the amygdala resulted in amygdaloid enlargement and glial activation

**Figure 5:**
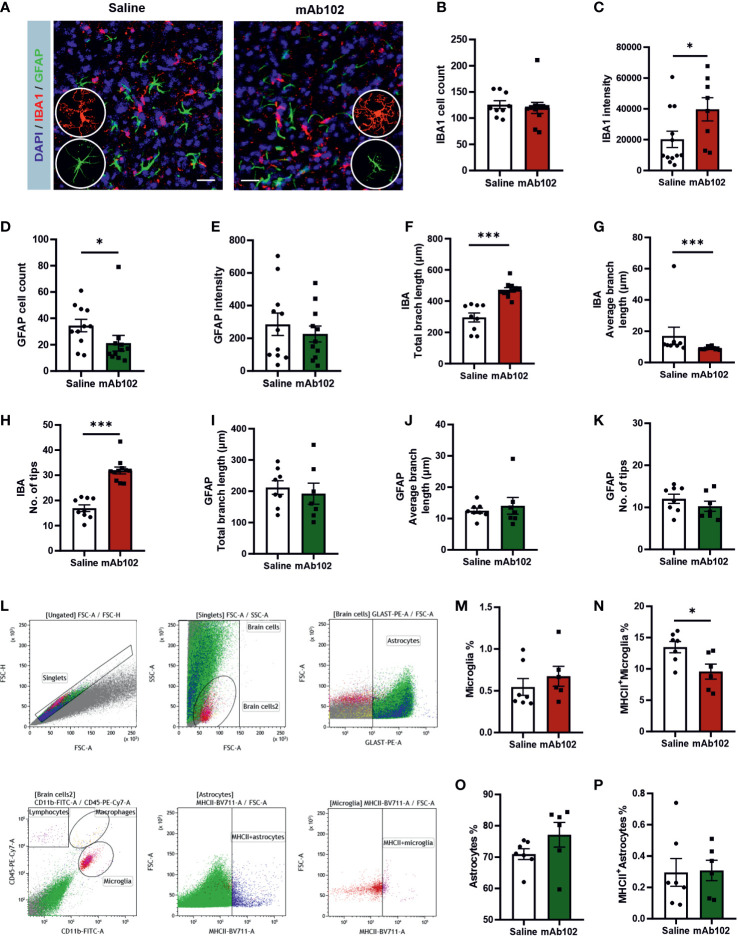
Quantification of amygdaloid width and glial responses in saline- and mAb102-injected mice. **(A)** IBA1 and GFAP immunostainings for microglia and astrocytes, respectively, are shown in immunohistochemical images. Scale bar = 10 μm. Zoomed images showing morphologies of microglia and astrocytes are inserted. **(B–E)** IBA1^+^ and GFAP^+^ cell number and intensity were quantified (*n*=11+11 slices from 3 + 3 mice). **(F–K)** Morphological parameters of IBA1^+^ (*n*=9+11 slices from 3 + 3 mice) and GFAP^+^ (*n*=8+7 slices from 3 + 3 mice) cells were quantified, including total branch length, average branch length, and number of tips. Microglia were more ramified and had increased IBA1 whereas astrocytes were less responsive in mAb102 group than their counterparts in saline group. See also **Table S4**. **(L)** Gating strategy for flow cytometry is shown. Debris and cell aggregates were excluded using correlation of FSC area vs height. For visual comparison, astrocytes and microglia were colored and identified as GLAST^+^ and CD45^+^CD11b^+^ cells, respectively. **(M–P)** The main populations of astrocytes, MCHII^+^ astrocytes, microglia and MCHII^+^ microglia are shown, from which the population of MCHII^+^ microglia underwent significant reduction in mAb102 group (*n*=7 mice) compared to saline group (*n*=6 mice). *p<0.05; ***p<0.001 (Student’s t-test, or Mann-Whitney U test).

Measuring amygdaloid size in MRI images (**Figure 4F**), we found the amygdaloid width was increased in mAb102 group compared to saline group (*p*=0.006; **Figure 4G**) (*n*=4+5), which stayed significant after normalization with the brain width (*p*=0.011; **Figure 4G**). Staining of brain sections with IBA1 and GFAP (**Figure 5A**) (*n*=3+3) showed that although the number of IBA1^+^-microglia had no group difference (**Figure 5B**), the average IBA1 intensity was higher in mAb102 group (*p*<0.05; **Figure 5C**). In comparison, the number of GFAP^+^-astrocytes was decreased in mAb102 group (*p*<0.05; **Figure 5D**), while the average GFAP intensity showed no group difference (**Figure 5E**).

Interestingly, microglia in mAb102-treated mice were over-ramified, showing longer total branch length, shorter average branch length and more tips (all *p*<0.001; **Figures 5F–H**; **Table S4**). Astrocytic morphology didn’t show striking differences in these parameters (**Figures 5I–K**) but displayed shorter length of inner branches in mAb102 group (*p*=0.034; **Table S4**), hence a tendency of de-ramification.

Moreover, flow cytometric analysis of amygdaloid tissues (**Figure 5L**) (*n*=7+7) showed that in microglia, while the total population did not differ (**Figure 5M**), the percentage of MHCII+-microglia was decreased in mAb102 group (*p*=0.026; **Figure 5N**). By contrast, total astrocytes and MHCII^+^ astrocytes did not change in mAb102 group (**Figure 5O, 5P**). We also measured the cytokines *Il1b, Il6, and Il10* mRNA levels but did not find significant differences in them between mAb102 and saline groups (data not shown).

## Discussion

We demonstrated that in FES-hs patients, blood *PLXNB2* mRNA level was the lowest compared to FES-ls patients and HCs; and lower stress level and larger amygdaloid size were associated with higher *PLXNB2* mRNA level in FES-hs patients. Plxnb2 was enriched in glial cells and dampened by CUS in the mouse brain. Furthermore, Plxnb2-inhibition in the amygdala induced anxiety and microglial activation in mice. These results altogether suggest that PLXNB2 may provide a glia-mediated protective mechanism sensitized in the amygdala for stress-coping, which may be dysregulated in schizophrenia.

Larger amygdala usually indicates stronger functional connections with other limbic structures and greater stress-coping capability and emotional intelligence normally (35). Indeed, in common individuals, increased levels of perceived stress were shown to be associated with enlarged amygdala and anterior hippocampus (36). As our current data supported, schizophrenia including FES patients have smaller amygdala, however, suggesting inefficient stress-coping (37–39). Dystrophy of the limbic structures could be due to elevated cortisol levels in FES patients, as we previously observed in schizophrenia patients’ urine samples (11). Besides, glucocorticoids are well-known to induce microglial activation and neuroinflammation that in turn exacerbate anxiety and depression (7, 23).

In our results, FES-hs patients showed stronger amygdala-dependency for stress perception as compared to HCs suggests biased stress-coping brain circuitry that was differentiated from HCs. This is also substantiated by that the caudate and thalamus were significantly bigger in these patients than HCs, indicative of their higher demand of energy needed for somatosensory processing during stress perception and handling. Corroboratively, their average PANSS-G score was higher and negatively correlated with the thalamic size, demonstrating thalamic involvement in psychopathology of this group of patients. Furthermore, the hippocampal and amygdaloid sizes were positively associated with PSS in HCs but not FES-hs patients.

By contrast, FES-ls patients showed no correlation of perceived stress with *PLXNB2* expression or amygdaloid size and had the smallest amygdala. Additionally, their hippocampi were smaller than those of FES-hs patients and were positively correlated with PANSS-N scores, suggesting impaired connection of limbic regions and cognition-dependent emotional processing in this unique group of patients. We hence propose that FES-ls patients reflect a different stress trait compared to FES-hs patients. Corroborating this suggestion, a recent study on autism found that autistic children with autism-distinct atypic anxiety had significantly slower amygdaloid growth and smaller amygdaloid size than autistic children with DSM-typic anxiety or non-autistic children (40).

Plxnb/Sema4 family is a key regulator in brain developmental processes including angiogenesis (41, 42), axonal growth (43, 44), differentiation of neurons (45, 46), and synaptic formation (47, 48). Alterations in all these developmental processes are known to contribute to ontogenesis of schizophrenia (1, 2). PLXNB2 may hence contribute to schizophrenia pathophysiology already at the developmental stage. Supportively, Paldy et al. recently demonstrated that Plxnb2 regulated fear memory *via* synaptic neurotransmission and remodeling using *Plxnb2*-deficient mice (48). This preclinical evidence also corroborates a protective role of Plxnb2 in stress regulation, as aberrant fear behavior is a core stress-related symptom in many psychiatric disorders including schizophrenia. Since what we used here were adult mouse models with relatively acute effects, such as in the case of CUS and mAb microinjection, it doesn’t reveal schizophrenia-related neurodevelopmental changes in which Plxnb2 might be involved. Nevertheless, our CUS model could reflect the relatively acute stress response of schizophrenia patients, since PSS scores represent recently experienced stress perception by patients (i.e., feelings within the past month at the time of the questionnaire).

We further observed glial enrichment of Plxnb2 in the adult mouse brains and decrease of Plxnb2 expression in astrocytes and nonglial cells after CUS, which was consistent with our clinical observation that *PLXNB2* was expressed at the lowest level in blood cells of FES patients with high stress as compared to the other two low stress groups. Importantly, blocking of Plxnb2 in the amygdala induced anxiety and amygdaloid enlargement in otherwise normal mice. Furthermore, although no overt astrocytosis and microgliosis were seen in the amygdala, microglia showed significantly enhanced IBA1 expression and over-ramification after Plxnb2-blocking.

Supporting microglial function of Plxnb2, a recent study discovered that microglia-specific Plxnb2 controlled neuroinflammation thereby restricting wound size in a mouse spinal cord injury model (18). It is noteworthy that MCHII^+^-microglia were decreased in mAb102 group, indicating a neuroprotective role of this subgroup of microglia. Aligned with this, our recent study found that CUS increased hippocampal MCHII^+^-microglia in female mice that were more resilient to stress-induced anxiety as compared to male mice (32). Additionally, PLXNB2 is a functional receptor for ANG that promotes angiogenesis and neurite outgrowth (49), sequelae that microglia closely monitor as well (50). Moreover, Paldy et al. previously demonstrated that Plxnb2 regulated development of peripheral somatosensory neurons and their nociceptive function in persistent inflammatory pain in mice (17).

Stress-induced over-ramification of microglia has been constantly observed by others and us (7, 24). Ramification of microglia usually indicates enhanced surveillance of the changing surrounding environment, such as changes in neurotransmission underlying anxiety-like behaviors (7, 23). In our experiments, it is plausible that mAb102 directly affected microglia-derived Plxnb2, but whether it may also affect neurons, as Paldy et al. demonstrated (48), which then indirectly induce microglial morphological changes and anxiety behavior, is considerable. Besides, since astrocytes are also important for brain and behavior, and dysfunctional astrocyte-microglia crosstalk contributes to microglial activation and neuroinflammation pertaining to pathogenesis in schizophrenia (20–22). In this sense, although we did not observe overt changes in number and morphology of astrocytes by mAb102, we did find reduced GFAP+astrocytes and therefore the potential contribution of astrocytic Plxnb2 to schizophrenia and stress disorders is worth pursuing more carefully in the future.

To condense, our study raises an idea of amygdaloid microglial activation in contribution to stress and anxiety, which may be leveraged by PLXNB2, and reveals a novel cellular and molecular mechanism in schizophrenia with different stress trait. It should however be cautioned that our study has several limitations, such as the small sample size in our clinical preclinical cohorts, the cross-sectional nature of our clinical study design, and the lack of mAb control group and depiction of potential neuronal/other glial changes in animal experiments. Nevertheless, our results warrant further research on PLXNB2 and its ligands in psychiatric disorders in the future.

## Data availability statement

The datasets presented in this study can be found in online repositories. The names of the repository/repositories and accession number(s) can be found below: European Nucleotide Archive (ENA) with the accession number: PRJEB53454.

## Ethics statement

The studies involving human participants were reviewed and approved by Institutional Ethical Committee of Beijing Huilongguan Hospital. The patients/participants provided their written informed consent to participate in this study. The animal study was reviewed and approved by Estonian National Board of Animal Experiments.

## Author contributions

FLX: animal experiments, data analysis, and paper writing. LYa: animal experiments and data analysis. YL, FF, HD, and MG: patient recruitment, clinical rating, neuroimaging, and sample collection. AK: amygdala microinjection. KC: flow cytometry. IH and KS: animal MRI and raw data analysis. GFH and LY: mAb102 antibody creation and verification. AZ and LEH: project funding and manuscript evaluation. LT and YT: project design, funding, data analysis, and manuscript revision. All authors have contributed to and have approved the final manuscript.

## Funding

This work was supported by the National Natural Science Foundation of China grants 81761128021 and 81771452, the National Institute of Health grants R01MH112180 and R01HL135160, the Estonian Research Council-European Union Regional Developmental Fund Mobilitas Plus Program No. MOBTT77, and the Estonian Research Council personal research funding team grant project No. PRG878 and No. PRG1473.

## Conflict of interest

LEH has received or plans to receive research funding or consulting fees on research projects from Mitsubishi, Your Energy Systems LLC, Neuralstem, Taisho, Heptares, Pfizer, Luye Pharma, Sound Pharma, Takeda, and Regeneron. None was involved in the design, analysis, or outcomes of the study.

The remaining authors declare that the research was conducted in the absence of any commercial or financial relationships that could be construed as a potential conflict of interest.

## Publisher’s note

All claims expressed in this article are solely those of the authors and do not necessarily represent those of their affiliated organizations, or those of the publisher, the editors and the reviewers. Any product that may be evaluated in this article, or claim that may be made by its manufacturer, is not guaranteed or endorsed by the publisher.

## Abbreviations

CPZ, Chlorpromazine; CUS, chronic unpredictable mild stress; CTQ, childhood trauma questionnaire; DEGs, differentially expressed genes; EPM, elevated plus maze; FDR, false discovery rate; FES, first-episode schizophrenia; GO, gene ontology; HC, healthy controls; ICV, intracranial volume; MRI, magnetic resonance imaging; OFT, open field test; PANSS-T/P/N/G, positive and negative symptom scale-total/positive/negative/general score; PLXNB2, plexin b2; PSSsum, perceived stress scale summation score; RNA-seq, RNA sequencing; SEMA, semaphorin.
