# Supplementary material for: Glial receptor PLXNB2 regulates schizophrenia-related stress perception via the amygdala
Source: Front Immunol. 2022 Oct 17;13:1005067. doi: 10.3389/fimmu.2022.1005067 (PMC9619215; doi:10.3389/fimmu.2022.1005067)
Supplement: Supplementary file 1 [file DataSheet_1.pdf]

Glial Receptor PLXNB2 Regulates Schizophrenia-related Stress Perception Via the Amygdala

Fang-Ling Xuan[1†], Ling Yan[1†], Yanli Li[2], Fengmei Fan[2], Hu Deng[2], Mengzhuang Gou[2], Keerthana Chithanathan[1], Indrek Heinla[1], Liang Yuan[3], Kadri Seppa[1], Alexander Zharkovsky[1], Anti Kalda[1], L. Elliot Hong[4], Guo-Fu Hu[3], Yunlong Tan[2#], Li Tian[1#]

Supplementary materials and methods

Participants

FES Patients (n=101) recruited for this study were from the Beijing Hui Long Guan Hospital. Patients were diagnosed schizophrenia according to the Structured Clinical Interview for DSM-IV (SCID), as confirmed independently by two psychiatrists. Inclusion criteria were: (1) 18-54-year-old Han Chinese; (2) illness duration ≤3 years (< 1 year on average); and (3) un-medicated or <2 weeks of anti-psychotic medication at the time of blood draw.

HC participants (n=49) matched for age and sex were recruited from local community through advertisements. Complete medical histories of HCs were collected, and physical examinations were conducted for all participants to identify those with chronic medical conditions. Candidates previously diagnosed with an Axis I psychiatric disorder, experienced substance abuse or dependence within the previous six months, with a history of autoimmune or other significant medical conditions, or received anti-inflammatory medications were excluded.

All participants provided written informed consent. The study was approved by the Institutional Ethical Committee of Beijing Huilongguan Hospital.

Demographic and clinical measures

Participants' past traumatic experiences were evaluated by Childhood Trauma Questionnaire (CTQ), a 29-item self-reported questionnaire of a retrospective measure encompassing five factors: physical abuse, emotional abuse, sexual abuse, physical neglect, and emotional neglect. Each factor has five corresponding items with five possible responses, ranging from 1 (never) to 5 (always), with the total overall trauma score ranging from 25 to 125, where higher score means more severe trauma [24]. Reliability and validity in general Chinese population and Chinese patients with mental disorders have been demonstrated [25].

Participants’ stress levels were evaluated based on Perceived Stress Scale (PSS), a 14-item self-reported questionnaire measuring feelings and thoughts during the last month. Each item has five possible responses, ranging from 0 (never) to 4 (very often), where higher score indicates higher perceived stress [26]). Reliability and validity in general Chinese population and Chinese patients with mental disorders have been demonstrated [27].

Whole blood collection and RNA-seq

Patient’s whole blood (5ml) was collected at 7-9am after overnight fasting using PAXgeneTM blood RNA tubes (Applied Biosystems). Tubes were shaken vigorously for at least 10 seconds (s) after sampling and immediately stored at -80°C. Total RNAs were extracted using Mag-MAXTM for Stabilized Blood Tubes RNA Isolation Kit (Applied Biosystems) following the manufacturer’s instructions. RNAs were quantified and assessed for purity by optical density ratios of 260nm/280nm and 260nm/230nm using NanoDrop spectrophotometry (ThermoFisher). RNA samples (1μg) were immediately sent to the Beijing Genomics Institute (BGI) for mRNA (after globin mRNA removal) sequencing on the BGIseq-500 platform. Quality controls on RNA samples (RIN/RQN⩾7.0, 28S/18S⩾1.0) were confirmed by BGI. Clean data of at least 4Gb (20M clean reads) per sample were collected.

RNA-seq data analysis

Data quality control and gene expression analysis were done on the Galaxy and NetworkAnalyst platforms [28] using EdgeR. Data with variance percentile rank <15% and counts <4 were filtered out. Counts per million reads were transformed and normalized. Log2 fold changes (Log2FC) with the significance of p values adjusted by Benjamini-Hochberg’s false discovery rate (FDR<0.05) were calculated for differentially expressed genes (DEGs) in group-wise comparisons among FES-hs, FES-ls patients and HCs.

MRI acquisition and processing

Brain structural MRI data were acquired using a Siemens Prisma 3.0T MRI scanner with a 64-channel head coil, located at the Beijing Huilongguan Hospital Magnetic Resonance Scanner Center. Foam pads were used to minimize head motions. Sagittal three-dimensional magnetization prepared rapid acquisition gradient echo (MPRAGE) was used to collect each participant’s anatomical data: repetition time (TR)/echo time (TE)/inversion time (TI)=2530/2.98/1100ms, flip angle (FA)=7º, field of view (FOV)=256×224mm^2, Pixel/gap size=1/0mm, matrix size=256×224bit. After scanning, two radiologists evaluated image

quality and if there were significant artefacts, images were recollected. After imputing corresponding internal anatomical instructions, 70 Desikan-Killiany (DK) atlas-based cortical and subcortical regions were extracted and data were processed with FreeSurfer software (http://surfer.nmr.mgh.harvard.edu) [29] following the ENIGMA pipeline, e.g., region-by-region visual checking and removal of incorrect values for brain segmentations (http://enigma.usc.edu/protocols/imaging-protocols). Bi-hemispheric regional areas and thicknesses were measured, regional and intracranial volumes (mm^3) were calculated, and no data were excluded.

Animals and chronic unpredictable stress (CUS)

Fourteen 8-week-old C57BL/6NTac male mice (Taconic) were randomly assigned into two groups (Control and CUS, n=7 per group) and group-housed with free access to food and water under a standard 12 hours (h) light-dark cycle. Mice were exposed to a variable sequence of 7 mild and unpredictable stressors once per day for 5 consecutive weeks [30]. The 7 stressors were: stroboscopic light overnight 1/s; rat odor and isolation overnight exposure; restraint for 2h; wet bedding and cage tilting at 45° overnight; food and water deprivation overnight; flipped light/dark exposure; and forced swimming at 18°C for 10min. All stressors were randomly scheduled and changed daily to sustain an unpredictable procedure. Animal experiments were done under license No. 171 issued from the Estonian National Board of Animal Experiments.

Animal brain MRI acquisition

Scanning was conducted in the University of Tartu Laboratory Animal Centre with 94/20 Bruker BioSpec small animal MRI. Microinjected mice (n=4+5) were anesthetized with 4% isoflurane (in medical air at 1.5l/min). During scanning body temperature and breathing rate were monitored and kept stable (around 36°C and 80-100bpm) using an animal bed integrated with circulating warm water and inhalation anesthetic of 1.5~2.5% isoflurane. 34 coronal slices were obtained using a T2-weighted high-resolution sequence with the following scanning parameters: FoV=20x20mm, TR=3585ms, TE=33ms, Image size=250x250 pixel, slice distanc=0.25mm, imaging time=12min (total time approximately 30min per animal).

The amygdaloid width was measured from the tip of the substantia nigra to the cortical edge at ~140° of angle from the horizontal line on slices of ca. -3mm~+2.75mm of the anterior commissure and normalized against the broadest brain width at ca. -2.97mm of the anterior commissure per animal using ImageJ.

PLXNB2 mAb production

PLXNB2 mAbs were produced cell immunization method. Briefly, 293 cells (ATCC, CRL-1573) expressing the full length of human PLXNB2 gene were used to immunize Balb/c mice. Hybridoma cells were generated and mAbs were purified by Protein A/G affinity chromatography. The clone 162A3C1 (mAb102) was used for intra-amygdaloid microinjection.

Analysis of mAb102 binding to and blocking of human and mouse PLXNB2

CHOK1 cells (ATCC, CCL-61) transfected with the full length of human and mouse PLXNB2 were maintained in F-12K medium plus 10% fetal bovine serum (FBS, Gibco). Cells were collected and centrifuged at 200g for 5 minutes (min) at room temperature (RT), washed once with phosphate-buffered saline (PBS)+2%FBS and re-suspended at a density of 3x106 cells/ml, transferred to 96 well plates in 100µl (3x105 cells/well). The plates were centrifuged at 300g for 5min at RT. For binding assay, mAb102 serially diluted in 100µl PBS+2%FBS was added to the corresponding wells and incubated at 4°C for 60min. The plates were then washed twice. AlexaFluor488-conjugated donkey anti-mouse IgG (H+L) (#R37114, Invitrogen, 1:1000) was added to each well and incubated at 4°C for another 60min. For functional blocking assay, each CHOK1-hPLXNB2-containing well was added with mixtures of 50µl of serially diluted mAb102 and 50µl of biotin-ANG (0.25µg/well) or biotin-SEMA4C (0.25µg/well) and incubated at 4°C for 60min. The plates were then washed twice with PBS+2% FBS. AlexaFluor488-conjugated streptavidin (#S32354, Invitrogen, 1:1000) were added to each well and the plates were incubated at 4°C for another 60min. The plates were then washed twice, and cells were re-suspended in 100µl/well PBS and analyzed by a flow cytometer BD FACSCanto II (BD Biosciences).

Flow cytometry isotype controls

Corresponding isotype control antibodies were IgG2b-PE (#400907), IgG2b-BV421 (#400639), IgG2a- BV650 (#400265), and IgG2b-APC (#400611) for CUS experiment, as well as IgG2b-AlexaFluor488 (#400625), IgG2b-PE/Cy7 (#400617), IgG2a-PE (#400211), and IgG2b-BV711 (#400653) for microinjection experiment.

Immunohistochemistry (IHC)

Microinjected mice (n=3+3) were deeply anesthetized with ketamine/xylazine and transcardially perfused with PBS and 4% paraformaldehyde (PFA). The brains were removed and post-fixed in 4% PFA at 4°C for 1d, followed by PBS washing and 30% sucrose for

cryoprotection, and were stored at -80°C before cryosectioning at -20°C. Coronal sections of 40μm-thickness were washed in PBS for 5min in 3 times with gentle rotation, followed by 15min incubation in 0.5% triton X. After PBS washings, slices were incubated with primary antibodies including rabbit anti-IBA1 (#SKL6615, Wako, 1:500) and mouse anti-GFAP conjugated with AlexaFluor488 (#53-9892-82, eBioscience, 1:500) in PBS+10% goat serum+1% bovine serum albumin+ 0.3M Glycine overnight at 4°C. Afterwards, slices were washed in PBS and incubated with goat anti-rabbit IgG H&L-AlexaFluor568 antibody (#ab175471, Abcam, 1:500) for 2h at RT, and then in 0.1μg/ml DAPI (#ACRO202710100, VWR) for 5min. Slices were washed in PBS and finally mounted to glass slides with Fluoromount™ aqueous mounting medium (#F4680, Sigma-Aldrich). (For details see supplementary materials)

Microscopic imaging and quantification

A FV1200MPE laser scanning microscope (Olympus) was used to image IBA1+ and GFAP+ cells with 10× and 60× objective lenses. All images were in 800×800 pixels, with a scanning velocity of 8.0 pixel/μm. For quantification of cell number and fluorescent intensity, images were taken with zoom in two times through a 10× objective lens. Z-stack images (step=0.5μm, depth=30μm) were taken for cell morphology. A "SNT" package in ImageJ was used to reconstruct cells in 3D and "Sholl analysis" was used to acquire the ramification index, while the other parameters were acquired through the "Quick measurements" of SNT, including total branches, average branch length, number and length of primary branches, inner branches, and tips. A total of ~33 microglia and ~12 astrocytes per group were measured.

Tables S1-S4: xlsx files

Figures S1-S4

Figure S1

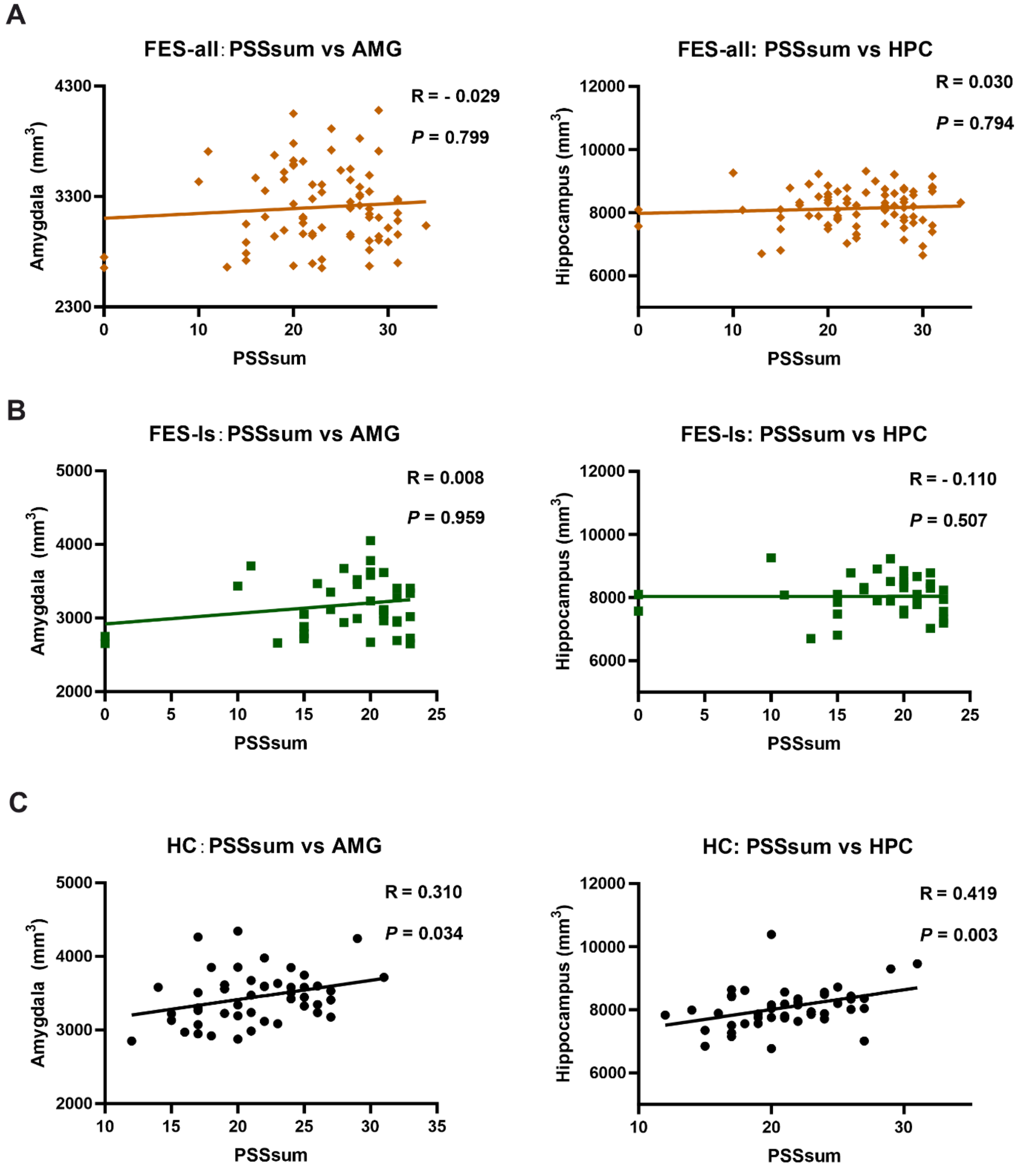

Figure S1. Correlations of sizes of amygdala and hippocampus with PSS scores in FES patients and HCs. Correlations in (**A**) all FES patients (n = 78), (**B**) FES-ls patients (n = 39)

and (**C**) HCs (n = 47) highlighted the positive relationship of the amygdala with PSS in HCs. (Spearman's correlation). AMG: amygdala; HPC: the hippocampus.

Figure S2

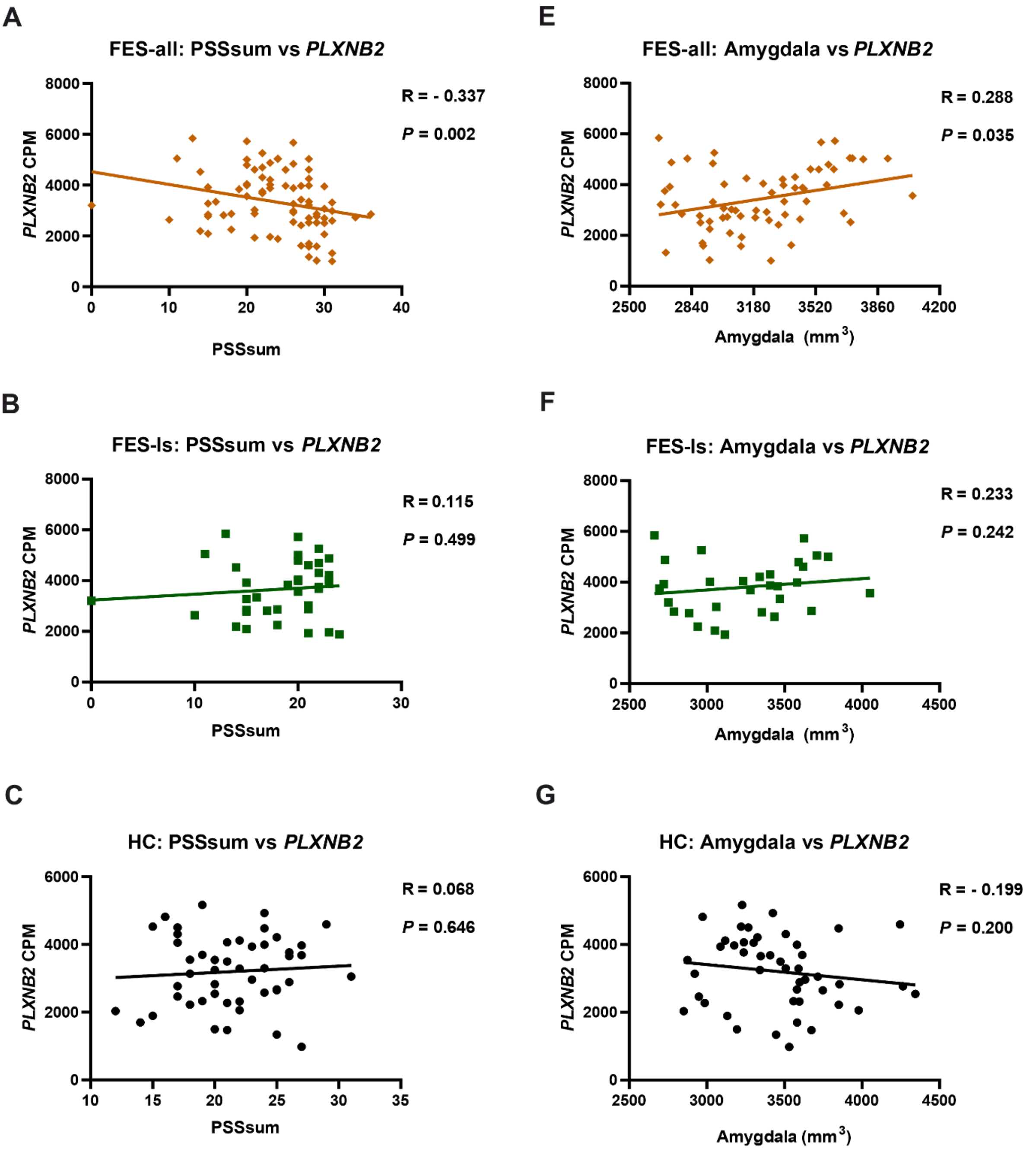

Figure S2. Correlation of Blood *PLXNB2* levels with PSS scores and amygdaloid size in FES patients and HCs. Correlations of *PLXNB2* with PSSsum (**A-C**) (Spearman's n = 81, 37, 48, respectively) and with amygdaloid size (**E-G**) (Pearson's n = 53, 25, 41, respectively)

highlighted their significances in FES patients (**A** and **E**), respectively, controlled by age, sex, and education.

Figure S3

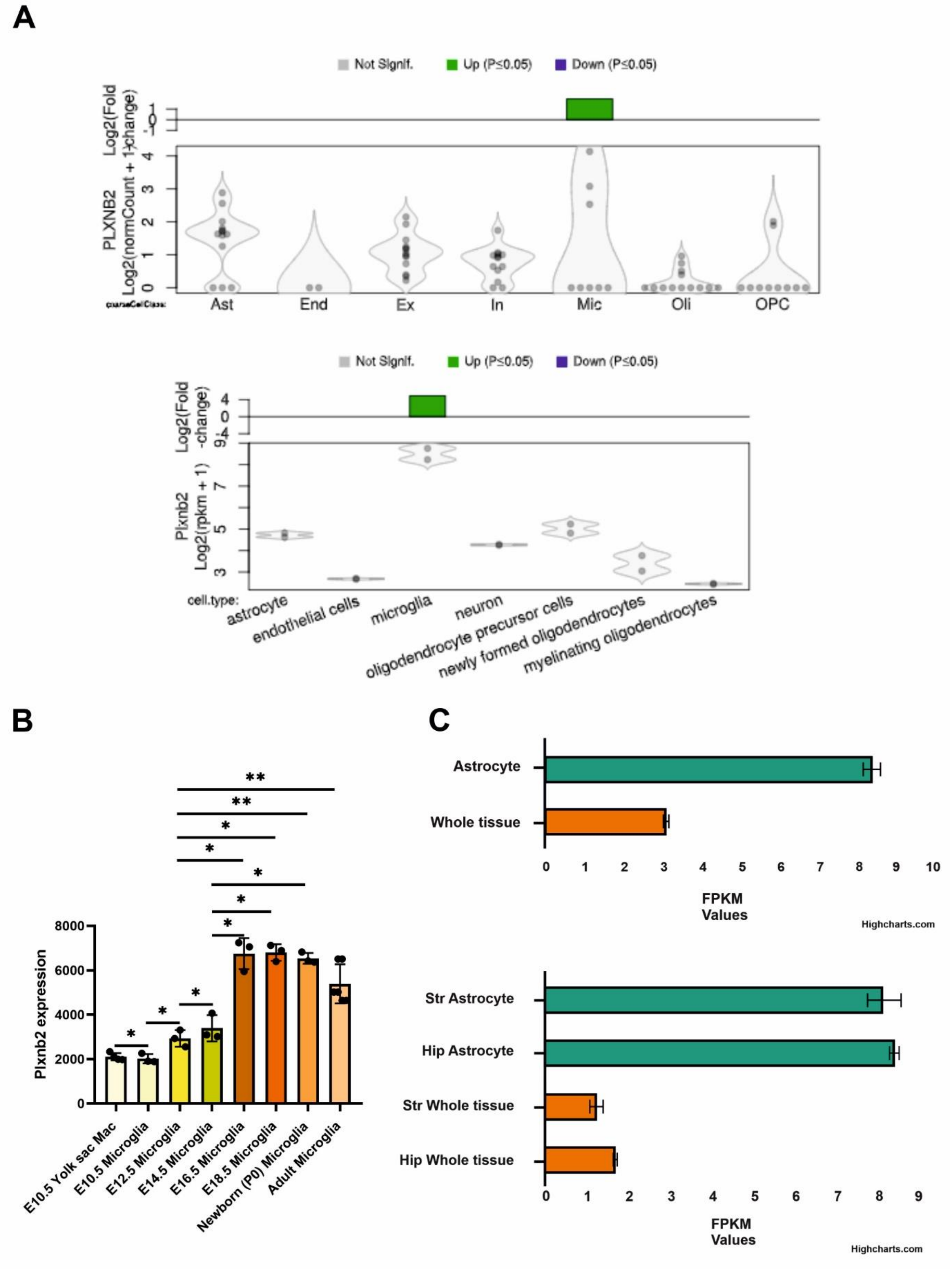

Figure S3. Expression of *Plxnb2* in human and murine microglia and astrocytes. (A) Microglia are the major cell type that express *PLXNB2* in the adult human (upper panel) and

mouse (lower panel) brains. Data were retrieved from The Myeloid Landscape 2 http://research-pub.gene.com/BrainMyeloidLandscape/BrainMyeloidLandscape2/. (**B**) *Plxnb2* expression in microglia increased significantly during E10.5 to E18.5 and peaked at E18.5. Afterward, it slightly reduced until its maturity. Data were derived from GSE#107129. (**C**) Expression of *Plxnb2* in adult murine astrocytes in the cortex (upper panel), striatum (Str, lower panel) and hippocampus (Hip, lower panel). Data were retrieved from human protein atlas (https://www.proteinatlas.org/) * *FDR* < 0.05; ** *FDR* < 0.01 (ANOVA).

Figure S4

A

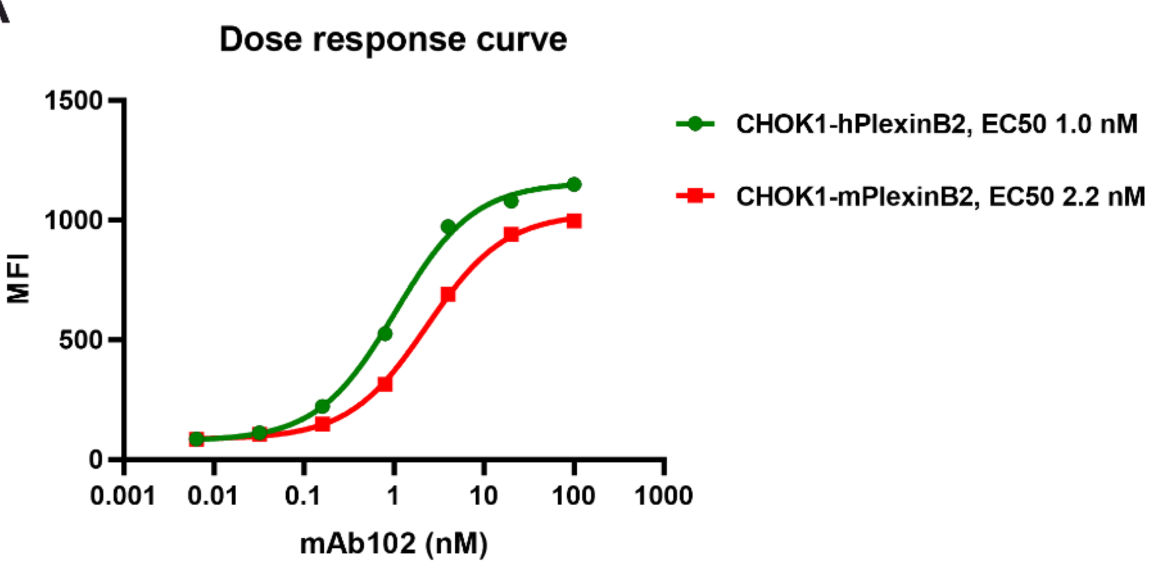

B

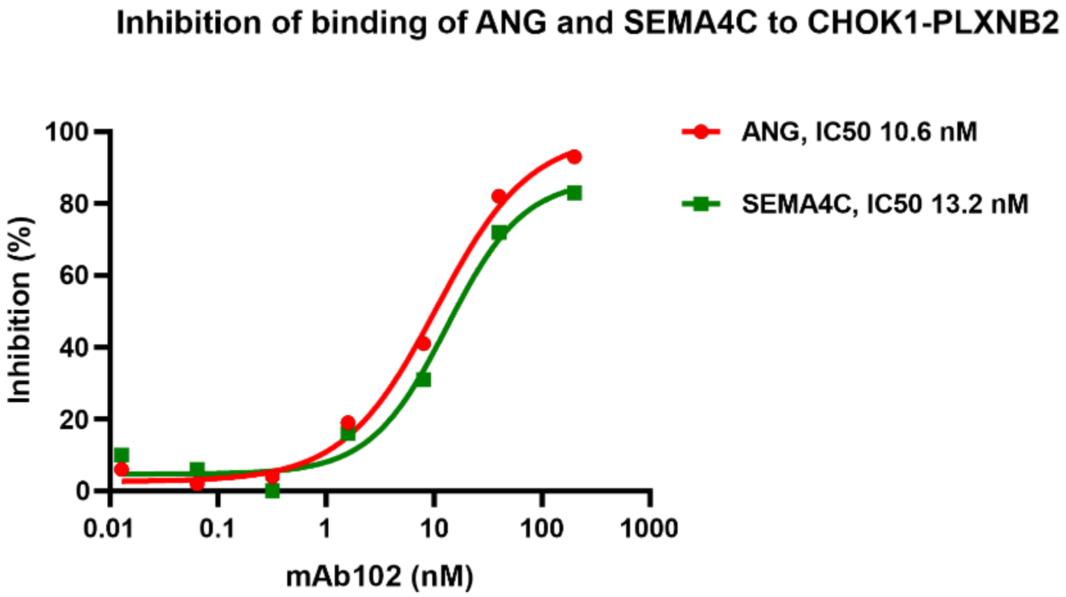

Figure S4. mAb102 affinity and inhibition of PLXNB2. (A) Dose response curves and calculated EC50 values of mAb102 binding to CHOK1-hPLXNB2 in comparison to CHOK1-mPlxnb2 cells. **(B)** Dose response curves of mAb102 inhibition of ANG and SEMA4C binding to CHOK1-hPLXNB2 cells.

Table S1.1. PSSsum vs Brain region_Spearman's correlations

R (*p, FDR*)	Amygdala	Caudate	Hippocampus	Thalamus
**FES-hs (n=39)**	**-0.415 (0.009, 0.04)**	0.011 (0.948, 0.95)	-0.269 (0.098, 0.20)	0.075 (0.648, 0.86)
**FES-ls (n=39)**	0.008 (0.959, 0.96)	-0.141 (0.392, 1.57)	-0.110 (0.507, 1.01)	0.012 (0.940, 1.25)
**HC (n=47)**	0.310 (0.034, 0.07)	0.214 (0.148, 0.15)	**0.419 (0.003, 0.01)**	0.287 (0.050, 0.07)

Table S1.2. PSSsum vs Gene_Spearman's correlations

R (*p, FDR*)	SEMA4A	SEMA4B	PLXNB2
**FES-hs (n=44)**	-0.219 (0.154, 0.23)	-0.169 (0.272, 0.27)	**-0.490 (0.001, 0.003)**
**FES-ls (n=37)**	0.062 (0.715, 1.07)	0.041 (0.811, 0.81)	0.115 (0.499, 1.50)
**HC (n=48)**	0.032 (0.831, 0.83)	-0.089 (0.548, 1.64)	0.068 (0.646, 0.97)

Table S1.3. PLXNB2 vs Brain region_Pearson's partial correlations (controlled for gender, age, education)

R (*p, FDR*)	Amygdala	Caudate	Hippocampus	Thalamus
**FES-hs (n=28)**	**0.471 (0.009, 0.04)**	0.021 (0.910, 0.91)	0.303 (0.104, 0.21)	-0.169 (0.371, 0.49)
**FES-ls (n=25)**	0.233 (0.242, 0.48)	-0.076 (0.706, 0.71)	-0.185 (0.355, 0.47)	-0.423 (0.028, 0.11)
**HC (n=41)**	-0.199 (0.200, 0.80)	-0.167 (0.285, 0.57)	-0.103 (0.509, 0.68)	0.022 (0.887, 0.89)

Table S1.4. PANSS vs Brain region_Pearson's partial correlations (controlled for gender, age, education)

	R (*p, FDR*)	Amygdala	Caudate	Hippocampus	Thalamus
**FES-hs (n=34)**	**PANSS-P**	-0.095 (0.581)	0.118(0.493)	-0.208 (0.223)	-0.250 (0.141)
	**PANSS-N**	-0.143 (0.405)	-0.114 (0.508)	0.136 (0.429)	0.131 (0.445)
	**PANSS-G**	0.130 (0.448)	0.001 (0.997)	-0.065 (0.706)	**-0.565 (0.0003, 0.001)**
	**PANSS-T**	-0.013 (0.938)	-0.006 (0.971)	-0.061 (0.723)	-0.413 (0.012)
**FES-ls (n=33)**	**PANSS-P**	-0.347 (0.041)	0.168 (0.335)	-0.140 (0.424)	0.187 (0.281)
	**PANSS-N**	0.070 (0.689)	0.091 (0.603)	**0.442 (0.008, 0.032)**	0.390 (0.021)
	**PANSS-G**	-0.256 (0.138)	0.047 (0.787)	-0.041 (0.816)	0.163 (0.349)
	**PANSS-T**	-0.255 (0.139)	0.129 (0.459)	0.112 (0.522)	0.338 (0.047)

Table S1.5. PANSS vs Gene_Pearson's partial correlations (controlled for gender, age, education)

	R (*p*)	SEMA4A	SEMA4B	PLXNB2
**FES-hs (n=39)**	**PANSS-P**	0.170 (0.287)	0.236 (0.137)	0.029 (0.857)
	**PANSS-N**	-0.024 (0.884)	-0.067 (0.676)	-0.049 (0.759)
	**PANSS-G**	0.219 (0.169)	0.290 (0.066)	-0.013 (0.937)

	**PANSSt**	0.181 (0.258)	0.227 (0.154)	-0.019 (0.904)
**FES-ls (n=31)**	**PANSS-P**	-0.201 (0.263)	-0.296 (0.094)	-0.154 (0.391)
	**PANSS-N**	-0.349 (0.047)	-0.392 (0.024)	-0.421 (0.015)
	**PANSS-G**	-0.218 (0.223)	-0.286 (0.107)	-0.124 (0.492)
	**PANSS-T**	-0.343 (0.051)	-0.433 (0.012)	-0.304 (0.084)

Table S2.1. PANTHER GO-Slim Molecular Function	Homo sapiens - R	Input (87(	Expected	Over/und(	Fold Enri(	Raw P-va	FDR
Olfactory receptor activity (GO:0004984)	145	2	61,32	-	0,03	7,38E-20	4,10E-17
**Transmembrane signaling receptor activity (GO:0004888)**	**669**	**174**	**282,9**	**-**	**0,62**	**1,70E-09**	**4,71E-07**
Signaling receptor activity (GO:0038023)	783	227	331,11	-	0,69	1,68E-07	2,33E-05
Molecular transducer activity (GO:0060089)	783	227	331,11	-	0,69	1,68E-07	3,11E-05
Structural constituent of ribosome (GO:0003735)	112	92	47,36	+	1,94	3,32E-06	3,68E-04
Catalytic activity (GO:0003824)	3989	1853	1686,83	+	1,1	2,06E-04	1,90E-02
G protein-coupled receptor activity (GO:0004930)	275	74	116,29	-	0,64	3,91E-04	3,10E-02

Table S2.2. String_GO:0004888_Protein domain (Smart)

Term description	Observed gene co	Backgrou	Strength	FDR	Matching proteins
Tyrosine kinase, catalytic domain	16	88	1,32	2,31E-14	PDGFRA,FLT4,IGF1R,EPHA1,(
Fibronectin type 3 domain	19	189	1,07	3,74E-13	EBI3,IGF1R,EPHA1,IL10RB,IFI
Immunoglobulin	20	328	0,85	2,85E-10	LAG3,PDGFRA,FLT4,IL18RAP
**ig-like, plexins, transcription factors**	**7**	**27**	**1,48**	**1,35E-07**	**MST1R,PLXND1,PLXNB1,PL**
**semaphorin domain**	**7**	**30**	**1,43**	**2,03E-07**	**MST1R,PLXND1,PLXNB1,PL**
G-protein-coupled receptor proteolytic site domain	7	33	1,39	2,99E-07	GPR133,EMR1,EMR2,GPR97,L
Immunoglobulin C-2 Type	13	211	0,85	4,14E-07	PDGFRA,FLT4,CSF1R,FGFR4,(
**domain found in Plexins, Semaphorins and Integrins**	**7**	**45**	**1,26**	**1,48E-06**	**MST1R,PLXND1,PLXNB1,PL**
Eukaryotic homologues of bacterial periplasmic substrate binding proteins	5	18	1,51	5,87E-06	GRIN3B,GRIK5,GRIN2D,GRIN
Ligated ion channel L-glutamate- and glycine-binding site	5	18	1,51	5,87E-06	GRIN3B,GRIK5,GRIN2D,GRIN
Domain present in hormone receptors	5	23	1,4	1,36E-05	VIPR1,LPHN1,CALCRL,BAI1,(
Ephrin receptor ligand binding domain	4	14	1,52	4,94E-05	EPHA1,EPHB3,EPHB4,EPHB6
Frizzled	4	18	1,41	0,00011	SMO,SFRP2,FZD1,FZD9
Immunoreceptor tyrosine-based activation motif	3	7	1,7	0,00021	CD3D,CD3E,CD3G
GS motif	3	7	1,7	0,00021	BMPR1A,TGFBR1,ACVR1B
Frizzled/Smoothened family membrane region	3	11	1,5	0,00054	SMO,FZD1,FZD9
Sterile alpha motif	4	86	0,73	0,0181	EPHA1,EPHB3,EPHB4,EPHB6
Immunoglobulin like	4	88	0,72	0,0184	PDGFRA,CSF1R,FCRL5,PECA
Link (Hyaluronan-binding)	2	14	1,22	0,0184	LYVE1,CD44
Furin-like repeats	2	18	1,11	0,0257	IGF1R,INSR
Domain abundant in complement control proteins	3	57	0,78	0,0289	GABBR1,IL2RA,IL15RA
Tumor necrosis factor receptor / nerve growth factor receptor repeats	2	26	0,95	0,0444	TNFRSF1A,TNFRSF1B

Table S2.3. FES vs HC Blood SEMA PLXN

EntrezID	logFC	AveExpr	t	P.Value	adj.P (FDR)	B	Symbols	Brain cell-specificity (b	Blood-specificity
**10509**	**0,48116**	**7,0599**	**7,1996**	**7,67E-12**	**1,28E-10**	**16,484**	**SEMA4B**	**Astrocytes, Microglia**	**Memory B-cell, naive B-cell**
**57715**	**0,87218**	**0,82089**	**6,3285**	**1,20E-09**	**8,25E-09**	**11,603**	**SEMA4G**	**Neurons**	**Memory B-cell**
**7869**	**0,90984**	**-0,59139**	**6,2643**	**1,71E-09**	**1,11E-08**	**11,26**	**SEMA3B**	**Astrocytes**	**Eosinophil**
**57556**	**0,87899**	**-0,01953**	**6,1329**	**3,51E-09**	**2,07E-08**	**10,567**	**SEMA6A**	**Astrocytes, Oligodendr**	**Low immune cell specificity**
**10500**	**0,49885**	**2,5883**	**6,014**	**6,68E-09**	**3,63E-08**	**9,9485**	**SEMA6C**	**Neurons**	**MAIT T-cell**
**10507**	**0,2991**	**8,8236**	**5,8648**	**1,48E-08**	**7,26E-08**	**9,1856**	**SEMA4D**	**Newly formed oligoden**	**Low immune cell specificity**
**56920**	**0,55724**	**-0,79397**	**4,0887**	**5,92E-05**	**0,00014105**	**1,2902**	**SEMA3G**	**Endothelia**	**T-reg**
**64218**	**0,2527**	**7,4994**	**4,0467**	**7,00E-05**	**0,00016472**	**1,1323**	**SEMA4A**	**Astrocytes, Newly forn**	**Neutrophil, myeloid DC, interm**
**54910**	**0,25372**	**4,9143**	**3,3916**	**0,000812**	**0,0016022**	**-1,1442**	**SEMA4C**	**Low brain cell specifici**	**Low immune cell specificity**
**10512**	**-0,37333**	**1,327**	**-3,3271**	**0,001015**	**0,0019683**	**-1,3487**	**SEMA3C**	**Neurons, Endothelia**	**Basophil**
**8482**	**0,24413**	**3,9081**	**3,1684**	**0,001731**	**0,0032234**	**-1,8369**	**SEMA7A**	**Endothelia**	**Eosinophil**
**9037**	**-0,43493**	**-2,0421**	**-2,5395**	**0,011732**	**0,01905**	**-3,553**	**SEMA5A**	**Newly formed oligoden**	**Plasmacytoid DC**
**223117**	**-0,3442**	**-4,1365**	**-2,4661**	**0,014359**	**0,022996**	**-3,7301**	**SEMA3D**	**Newly formed oligoden**	**Naive B-cell**
10501	0,17642	0,62171	1,3349	0,18317	0,23314	-5,8221	SEMA6B	Low brain cell specificit	Not detected in immune cells
10371	-0,22024	-3,0249	-1,2946	0,19669	0,24854	-5,8741	SEMA3A	Neurons	Non-classical monocyte
6405	0,082756	-4,2195	0,59416	0,55296	0,61438	-6,5257	SEMA3F	Neurons, Microglia, Enc	Not detected in immune cells
54437	-0,039201	-3,5301	-0,23831	0,81184	0,84678	-6,6721	SEMA5B	Astrocytes, Newly form	Not detected in immune cells
9723	-0,0093755	-4,2663	-0,06622	0,94726	0,95894	-6,698	SEMA3E	Newly formed oligodenc	Low immune cell specificity
**55558**	**0,68906**	**4,75**	**7,2565**	**5,43E-12**	**9,96E-11**	**16,818**	**PLXNA3**	**Low brain cell specifici**	**Low immune cell specificity**
**5361**	**0,43512**	**3,7722**	**6,3261**	**1,21E-09**	**8,34E-09**	**11,589**	**PLXNA1**	**Low brain cell specifici**	**Low immune cell specificity**
**5362**	**0,3704**	**2,7119**	**4,7031**	**4,32E-06**	**1,26E-05**	**3,7597**	**PLXNA2**	**Low brain cell specifici**	**Low immune cell specificity**
**5364**	**0,44706**	**0,92557**	**4,1625**	**4,39E-05**	**0,00010709**	**1,5711**	**PLXNB1**	**Low brain cell specifici**	**Not detected in immune cells**
**23129**	**0,20757**	**6,4812**	**3,728**	**0,000241**	**0,0005181**	**-0,01974**	**PLXND1**	**Neurons, Endothelia**	**Classical monocyte**
**23654**	**0,16178**	**7,5262**	**2,8926**	**0,00417**	**0,0072923**	**-2,6329**	**PLXNB2**	**Microglia**	**Non-classical monocyte, interme**
**91584**	**-0,20042**	**1,237**	**-2,2011**	**0,028679**	**0,043187**	**-4,328**	**PLXNA4**	**Neurons, Microglia, As**	**Plasmacytoid DC**
10154	0,13611	7,375	1,6115	0,10838	0,14595	-5,4226	PLXNC1	Astrocytes, Oligodendro	Neutrophil
5365	-0,12709	-0,32206	-1,0848	0,27911	0,33873	-6,1196	PLXNB3	Newly formed oligodenc	Plasmacytoid DC

Table S3.1. FES-hs (n = 44) vs FES-ls (n = 37) vs HC (n = 48)_3 groups comparison_RNAseq SEMA PLXN counts

EntrezID	LogFC.FS_hs.FS_ls	LogFC.FS_hs.HC	LogFC.FS_ls.HC	LogCPM	LR	P Value	Adj.P (FDR)	Symbols	Brain cell-specificity (brainrnaseq.org)
64218	-0,23679	0,18202	0,41881	8,0455	16,869	0,000217	0,0095	SEMA4A	Astrocytes, Newly formed oligodendroctyes
10509	-0,077342	0,25691	0,33426	7,7532	18,594	9,17E-05	0,0052	SEMA4B	Astrocytes, Microglia
54910	-0,063911	-0,071769	-0,007858	5,6466	1,2205	0,54321	0,7919	SEMA4C	Low brain cell specificity (endothelia)
10507	-0,066176	0,15556	0,22173	9,3901	9,6993	0,007831	0,081	SEMA4D	Newly formed oligodendroctyes, Myelinating oligodendroctyes, Microglia
57715	-0,057235	-0,015822	0,041414	1,8732	0,4899	0,78274	0,9165	SEMA4G	Neurons
5361	0,054011	0,10812	0,054108	4,4437	2,3558	0,30792	0,6153	PLXNA1	Low brain cell specificity
5362	-0,10188	0,077306	0,17918	3,3628	2,3892	0,30282	0,6107	PLXNA2	Low brain cell specificity: Endothelia, Astrocytes, Newly formed oligodendroctyes, Neurons
55558	0,054682	0,15634	0,10166	5,566	4,2669	0,11843	0,3812	PLXNA3	Low brain cell specificity: Astrocytes, Newly formed oligodendroctyes, Oligodendroctye precursor cells, Neurons
23654	0,0021485	0,088517	0,086369	7,9793	1,3944	0,49797	0,7691	PLXNB2	Microglia
10154	-0,071532	-0,071529	2,82E-06	7,9062	0,37766	0,82793	0,9338	PLXNC1	Astrocytes, Oligodendroctye precursor cells, Neurons
23129	0,15313	0,13463	-0,018495	7,0021	3,987	0,13622	0,4168	PLXND1	Neurons, Endothelia

Table S3.2. FES-hs (n = 44) vs FES-ls (n = 37) vs HC (n = 48)_3 groups comparison_1247 DEGs_pathway analysis

Pathway	Total	Expected	Hits	P.Value	FDR
**Anion transport**	**374**	**0,236**	**4**	**5,25E-05**	**0,043**
Organic acid transport	246	0,155	3	0,000392	0,107
Carboxylic acid transport	246	0,155	3	0,000392	0,107
Amino acid transport	143	0,0901	2	0,00342	0,701

FES-hs (n = 44) & FES-ls (n = 37) vs HC (n = 48) comparison_500 overlapping DEGs_pathway analysis_String

Term description	Observed gene count	Background gene c	Strength	FDR	Matching proteins
Colorectal cancer	12	85	0,79	0,00043	RALA,EREG,KRAS,PIK3R3,AK
Phospholipase D signaling pathway	14	145	0,62	0,0017	RALA,KRAS,RRAS2,PIK3R3,AK
Longevity regulating pathway - multiple species	8	61	0,76	0,0099	KRAS,PIK3R3,AKT3,HSPA6,AT
VEGF signaling pathway	8	59	0,77	0,0099	KRAS,PIK3R3,AKT3,SHC2,NRA
Natural killer cell mediated cytotoxicity	11	124	0,59	0,0113	KRAS,PIK3R3,SHC2,CASP3,PTF
Proteoglycans in cancer	14	195	0,5	0,0113	WNT6,KRAS,RRAS2,PIK3R3,AK
Signaling pathways regulating pluripotency of stem cel	11	138	0,54	0,0189	WNT6,KRAS,POU5F1,PIK3R3,A
ErbB signaling pathway	8	83	0,62	0,0303	EREG,KRAS,PIK3R3,AKT3,SHC
Endocrine resistance	8	95	0,57	0,0317	KRAS,PIK3R3,AKT3,SHC2,NRA
Ras signaling pathway	14	228	0,43	0,0317	RALA,RAP1B,KRAS,RRAS2,PIK
Longevity regulating pathway	8	88	0,6	0,0317	KRAS,PIK3R3,AKT3,APPL1,AT
Fc epsilon RI signaling pathway	7	67	0,66	0,0317	KRAS,PIK3R3,AKT3,FCER1A,N
Estrogen signaling pathway	10	133	0,52	0,0317	KRAS,PIK3R3,AKT3,SHC2,HSP
Prolactin signaling pathway	7	69	0,65	0,0317	LHB,KRAS,PIK3R3,AKT3,SHC2
Relaxin signaling pathway	10	130	0,53	0,0317	KRAS,PIK3R3,AKT3,SHC2,GNG
Renal cell carcinoma	7	68	0,65	0,0317	RAP1B,KRAS,PIK3R3,AKT3,PT
Acute myeloid leukemia	7	66	0,67	0,0317	SPI1,KRAS,PIK3R3,AKT3,TCF7
Chronic myeloid leukemia	7	76	0,6	0,035	KRAS,PIK3R3,AKT3,SHC2,PTP
Endometrial cancer	6	58	0,65	0,0392	KRAS,PIK3R3,AKT3,TCF7L1,N
Autophagy - animal	9	125	0,5	0,0395	KRAS,RRAS2,PIK3R3,AKT3,MF
Chemokine signaling pathway	11	181	0,42	0,0477	RAP1B,KRAS,PIK3R3,AKT3,SH
Cellular senescence	10	156	0,45	0,0477	KRAS,RRAS2,PIK3R3,AKT3,MF

FES-hs (n = 44) vs HC (n = 48)_comparison_329 non-overlapping DEGs_pathway analysis_String

Term description	Observed gene count	Background gene c	Strength	FDR	Matching proteins
Cytokine Signaling in Immune system	14	328	0,55	0,003	MT2A,LIF,LAMA5,TNFRSF13C,

FES-ls (n = 37) vs HC (n = 48)_comparison_681 non-overlapping DEGs_pathway analysis_String

Term description	Observed gene count	Background gene c	Strength	FDR	Matching proteins
Cell activation	60	1024	0,29	0,0097	GRN,UNC13D,ARSA,IL4,MAPK
Immune effector process	50	927	0,25	0,0245	GRN,UNC13D,ARSA,WASL,UN
Myeloid leukocyte activation	37	574	0,33	0,0245	GRN,UNC13D,ARSA,IL4,CNN2,
Myeloid cell activation involved in immune response	35	519	0,35	0,0245	GRN,UNC13D,ARSA,CNN2,ATF
Leukocyte activation involved in immune response	39	616	0,32	0,0245	GRN,UNC13D,ARSA,CNN2,ATF
Leukocyte mediated immunity	38	632	0,3	0,0245	GRN,UNC13D,ARSA,IL4,CNN2,
Myeloid leukocyte mediated immunity	35	519	0,35	0,0245	GRN,UNC13D,ARSA,CNN2,ATF
Cellular process	484	14652	0,04	0,0245	MAP4K5,FAM13B,GRN,KCNQ1
Vesicle-mediated transport	83	1699	0,21	0,0245	GRN,UNC13D,ARSA,BLOC1S6,I
Leukocyte degranulation	35	507	0,36	0,0245	GRN,UNC13D,ARSA,CNN2,ATF
Neutrophil degranulation	33	485	0,35	0,0245	GRN,UNC13D,ARSA,CNN2,ATF
Positive regulation of multi-organism process	30	394	0,4	0,0245	CAV2,UNC93B1,WDFY1,PTPLA
Leukocyte activation	52	894	0,28	0,0245	GRN,UNC13D,ARSA,IL4,CNN2,
Regulation of biological process	385	11116	0,06	0,0245	MAP4K5,FAM13B,GRN,KCNQ1
Regulation of cellular process	365	10484	0,06	0,0245	MAP4K5,FAM13B,GRN,KCNQ1
Biological regulation	402	11740	0,05	0,0245	MAP4K5,FAM13B,GRN,KCNQ1
Regulation of intracellular signal transduction	84	1764	0,2	0,0245	MAP4K5,FAM13B,P2RY10,GMI
Immune response	74	1560	0,19	0,0288	GRN,UNC13D,ARSA,RNF125,UI
Regulated exocytosis	40	691	0,28	0,0288	GRN,UNC13D,ARSA,BLOC1S6,(
Regulation of multi-organism process	38	653	0,28	0,0361	RNF125,CAV2,UNC93B1,WDFY
Regulation of metabolic process	240	6516	0,08	0,0377	MAP4K5,KCNQ1,MAPK13,PABI
Regulation of response to stimulus	154	3882	0,12	0,0412	MAP4K5,FAM13B,GRN,P2RY10
Immune system process	102	2370	0,15	0,0427	GRN,UNC13D,ARSA,RNF125,W
Regulation of cellular metabolic process	225	6082	0,09	0,0493	MAP4K5,MAPK13,PABPN1,RNI

FES-ls (n = 37) vs hs (n = 44)_comparison_6 non-overlapping DEGs_pathway analysis_String

Term description	Observed gene count	Background gene c	Strength	FDR	Matching proteins
Hemoglobin complex, and glycophorin a	2	19	2,71	0,0299	GYPB, MAPK8IP1P2, FAM83A,

Table S4. Saline vs mAb102_ Comparison of mophological parameters of IBA1 and GFAP cells

Group	Marker	Ramification index	Total brach length (µm)	Average branch length (µm)	No. of branches	No. of primary branches
**Saline**	**IBA1**	(2.773, 0.252)	(295.390, 28.580)	(17.041, 5.590)	(28.519, 2.540)	(4.680,0.337)
	**GFAP**	(1.959, 0.187)	(211.764, 21.589)	(12.520, 0.841)	(17.980, 2.059)	(5.917, 0.516)
**mAb102**	**IBA1**	0.418 (3.018, 0.150)	**< 0.001** (472.284, 14.286)	**< 0.001** (9.118, 0.278)	**< 0.001** (57.173, 2.750)	**0.001** (5.996, 0.285)
	**GFAP**	0.199 (1.625, 0.138)	0.636 (192.330, 33.472)	0.867 (14.077, 2.646)	0.289 (14.691, 2.140)	0.472 (5.452, 0.354)

Values represent p (Mean, SEM)

No. of inner branches	No. of tips	Length of primary branches (µm)	Length of inner branches (µm)	Length of terminal branches (µm)
(3.930, 0.350)	(16.926, 1.336)	(76.104, 7.120)	(37.670, 4.191)	(201.927, 18.476)
(3.146, 0.760)	(12.0417, 1.078)	(81.115, 9.326)	(25.354, 5.950)	(170.493, 15.650)
0.045 (4.868, 0.252)	< **0.001** (31.889, 1.387)	0.104 (95.238, 8.615)	0.259 (43.138, 1.900)	**0.005** (272.230, 8.246)
0.166 (4.833, 0.861)	0.290 (10.286, 1.190)	0.645 (89.608, 11.330)	**0.034** (48.246, 151.970)	0.125 (151.970, 28.401)
